# Discovery of genes affecting resistance of barley to adapted and non-adapted powdery mildew fungi

**DOI:** 10.1186/s13059-014-0518-8

**Published:** 2014-12-03

**Authors:** Dimitar Douchkov, Stefanie Lück, Annika Johrde, Daniela Nowara, Axel Himmelbach, Jeyaraman Rajaraman, Nils Stein, Rajiv Sharma, Benjamin Kilian, Patrick Schweizer

**Affiliations:** Leibniz-Institut für Pflanzengenetik und Kulturpflanzenforschung (IPK) Gatersleben, Corrensstrasse 3, 06466 Stadt Seeland, Germany; Current address: Syngenta Seeds GmbH, Zum Knipkenbach 20, 32107 Bad Salzuflen, Germany; Current address: The James Hutton Institute, Invergowrie, Dundee, DD2 5DA Scotland UK; Current address: Bayer CropScience SA-NV, J.E. Mommaertslaan 14, 1831 Diegem, Machelen Belgium

## Abstract

**Background:**

Non-host resistance, NHR, to non-adapted pathogens and quantitative host resistance, QR, confer durable protection to plants and are important for securing yield in a longer perspective. However, a more targeted exploitation of the trait usually possessing a complex mode of inheritance by many quantitative trait loci, QTLs, will require a better understanding of the most important genes and alleles.

**Results:**

Here we present results from a transient-induced gene silencing, TIGS, approach of candidate genes for NHR and QR in barley against the powdery mildew fungus *Blumeria graminis*. Genes were selected based on transcript regulation, multigene-family membership or genetic map position. Out of 1,144 tested RNAi-target genes, 96 significantly affected resistance to the non-adapted wheat- or the compatible barley powdery mildew fungus, with an overlap of four genes. TIGS results for QR were combined with transcript regulation data, allele-trait associations, QTL co-localization and copy number variation resulting in a meta-dataset of 51 strong candidate genes with convergent evidence for a role in QR.

**Conclusions:**

This study represents an initial, functional inventory of approximately 3% of the barley transcriptome for a role in NHR or QR against the powdery mildew pathogen. The discovered candidate genes support the idea that QR in this *Triticeae* host is primarily based on pathogen-associated molecular pattern-triggered immunity, which is compromised by effector molecules produced by the compatible pathogen. The overlap of four genes with significant TIGS effects both in the NHR and QR screens also indicates shared components for both forms of durable pathogen resistance.

**Electronic supplementary material:**

The online version of this article (doi:10.1186/s13059-014-0518-8) contains supplementary material, which is available to authorized users.

## Background

Plant-pathogen co-evolution has shaped a multifaceted innate immunity system triggered by the recognition of non-self-molecules via pathogen recognition receptors (PRRs) belonging to the family of receptor-like kinases (RLKs) [1]. These non-self-molecules known as pathogen-associated molecular patterns (PAMPs) or, more generally, microbe-associated molecular patterns (MAMPs) include conserved domains of proteins such as bacterial flagellin (flg22) or chitin fragments from fungal cell walls [2]. PAMP-triggered immunity (PTI) has been recognized as the most ancient type of plant defense sharing also components with the innate immunity system of vertebrate and invertebrate animals. Downstream of PRRs its molecular components include MAP kinases, WRKY transcription factors as well as an arsenal of downstream-responsive, (WRKY-regulated) genes encoding proteins that generate reactive oxygen species, reinforce, and break down plant and pathogen cell-walls, respectively, or catalyze the synthesis of pathogen-toxic compounds such as phytoalexins. On top of PTI plants can activate an effector-triggered immunity (ETI) response that is based on the direct or indirect recognition of avirulenve (Avr) effector molecules of some pathogen races by major R-genes encoding nucleotide-binding leucine-rich repeat (NB-LRR) proteins, and on the initiation of a very strong local defense response often culminating in host-cell death. One of the preferred targets of effectors are PRRs, which have been found to be guarded by several NB-LRR type or PRR-like proteins therefore also being involved in ETI [3]. Durable and broad-range non-host resistance (NHR) to virtually all races of non-adapted pathogens appears to be an important manifestation of PTI in many cases [4,5] although there is also experimental evidence that NHR can - at least in grass species - be mediated by as little as one major R gene recognizing an indispensable Avr effector [6]. Race-specificity of NHR QTL to non- or only partially adapted fungal pathogens has also been described, similar to QTL for host quantitative resistance (QR) that is another manifestation of PTI [7] and QR is also referred to as race-non-specific or horizontal resistance [8-10]. However, in contrast to the very robust NHR response, QR is often not very efficient suffering from effector-triggered susceptibility (ETS) brought about by small secreted proteins or peptides from adapted pathogens that are active in the plant apoplast or inside host cells [11]. The introgression of single major R genes usually confers strong protection against specific adapted pathogen races carrying the matching avirulence (*Avr*) effector genes, but the trait is often overcome by rapidly evolving new pathogen races with mutated *Avr* effectors acting in concert with other functionally redundant effectors. In principle, QR could also be mediated by partially functional (defeated) major R-genes weakly recognizing ubiquitous *Avr* effectors such as ECP1 or ECP2 [12], but molecular evidence for this type of interactions is scarce [13,14].

Barley (*Hordeum vulgare* ssp. *vulgare*) is an important crop plant and exhibits genetic variability determining to which extent it is successfully colonized by a given pathogen. This opens up the possibility to improve QR as a quantitative trait by introgressing and/or combining resistance-related alleles. Often, however, QR was found to be inherited by many QTLs making the trait difficult to handle in breeding practice due to complex crossing schemes, phenotype scoring ambiguities and linkage drag problems [9]. Knowing the genes that encode important QR components in crop plants would render targeted QR improvement by allele mining and gene marker-assisted as well as pathway-oriented introgression more efficient. One of the major diseases of barley is powdery mildew caused by the obligate biotrophic ascomycete fungus *Blumeria graminis* f.sp. *hordei* (*Bgh*) [15] that also fulfills several criteria of a model plant-pathogen interaction due to a large body of physiological, cellular, biochemical, and molecular information on changes in the host during compatible or resistant interactions [16-18]. Transient expression and gene-silencing assays such as transient-induced gene silencing (TIGS) in bombarded epidermal cells have been developed over the years and proven to be valuable tools for a better understanding of barley/powdery mildew interactions [19-21]. NHR of barley against non-adapted *formae speciales* or species of powdery mildew such as the wheat pathogen *B. graminis* f.sp. *tritici* (*Bgt*) efficiently blocks fungal penetration attempts at the epidermal cell wall and shares a number of genes with the QR pathway triggered by the adapted barley powdery mildew fungus [22-24]. Therefore, the discovery of important genes required for NHR may also reveal their importance in QR, besides opening up the fascinating option of replacing effector-suppressed defense factors by their non-host orthologues by wide crosses or in transgenic plants.

Here we describe a large-scale functional approach in barley for the identification of genes that are relevant for NHR and QR to *Bgt* and *Bgh*, respectively [23]. By using TIGS we screened three groups of host genes that were: (1) previously found to be upregulated in powdery mildew-attacked barley epidermal cells [19,21,25,26]; (2) belonging to selected multigene-families; or (3) localizing within the confidence interval of a meta-QTL for resistance to *Bgh* on chromosome 5H [9,27]. The data of QR modulation by RNAi constructs targeting the corresponding transcripts were combined with meta-data of transcript regulation, SNP or gene haplotype associations with QR to *Bgh*, co-localization of the RNAi target genes with QTL for resistance to *Bgh*, and copy number variation. As main result we present a first inventory of barley genes that are likely to play a role in broad-spectrum, durable resistance against powdery mildew fungi.

## Results

### Selection of gene groups

In total 1,274 TIGS constructs were bombarded into barley epidermal cells, which corresponded to 1,144 candidate target genes due to a certain number of redundant constructs targeting the same genes. In the case of redundant constructs those with the statistically most significant effects were selected for further analysis. After the bombardment, leaf segments were inoculated either with *Bgt* (NHR screening) or *Bgh* (QR screening). The sets of TIGS constructs used in both screens overlapped partially and targeted primarily transcripts found to be upregulated during host or non-host interactions of barley epidermis with *Bgh* or *Bgt*. (Table 1). Because the selection was based on preliminary transcript-profiling data (two out of four biological replicates) a number of putatively upregulated genes finally turned out to be not regulated. This resulted - together with TIGS constructs containing unintended target-gene sequences due to PCR artifacts or other errors - in a group of genes we referred to as ‘randomly selected’ in retrospect. A third group of entry genes, which were tested only in the QR screen, consisted of members of eight multigene families that have been described in barley or other plant species to contain important PTI or ETS components (see Table 2 for details of the QR screen). The fourth group consisted of 111 target genes that could be mapped to the confidence interval of a meta-QTL for QR to *Bgh* on the short arm of chromosome 5H. We were previously directed towards this meta-QTL by an association-genetic approach of candidate genes that revealed co-localization of several QR-associated genes within this region at genetic distances extending beyond local linkage disequilibrium [9,27-29]. Thus, the functional architecture of the resistance QTL on 5HS might be complex with more than one causative gene acting either independently or *in concerto* in different genotypes of the association-mapping panel.

**Table 1:** **Summary of the TIGS screens for candidate genes of non-host resistance (NHR) and quantitative host resistance (QR)**

**Screen**	**Tested in 1st round screen**		**Sign. TIGS effect** ^**a**^	
	**Constructs**	**Genes**	**With FDR 0.1**	**Without error 1 correction**
NHR only	260	230	1	6
QR only	582	513	41	86
NHR and QR	432	401	2	4
NHR all	692	631	3	10
QR all	1,014	914	43	90
Total^b^	1,274	1,144	44	96

^a^Number of target genes with significant TIGS effect (*P* <0.05; one-tailed Mann-Whitney test against empty-vector control for NHR screen; one-tailed *t*-test against median value of all tested constructs for QR screen). FDR for multiple testing-corrected significance thresholds was set to 0.1 (10%).

^b^NHR_only_ + (NHR and QR) + QR_only_.

**Table 2:** **Comparison of TIGS effects between candidate-gene groups for quantitative host resistance (QR)**

**Selection criterion**	**Family**	**Entry** ^**a**^	**Repeated** ^**b**^	**Significant TIGS effect** ^**c**^		
		**Genes (n)**	**Genes (n)**	**Genes (n)**	**%**	***P*** ^**d**^
Regulated	Multiple	345	112	34	9.9	0.084
Random	Multiple	87	33	4	4.6	-
Gene family	ABC transporter	90	19	8	8.9	0.372
	Cellulose-synthase like	22	15	5	22.7	0.016
	LysM-, LRR-RLK	35	10	7	20.0	0.013
	Class III Peroxidase	69	16	6	8.7	0.339
	Proteasome lid comp.	51	17	3	5.9	0.709
	U box-, RING-E3 ligase	36	7	4	11.1	0.231
	Sweet sugar transporter	12	12	4	33.3	0.007
	WKRY TF	58	16	7	12.1	0.116
QTL ci^e^	Multiple	109	31	8	7.3	0.554
Total		914	288	90	9.8	-

^a^Number of TIGS target genes for first screening round.

^b^Relative to the number of genes targeted by repeated bombardments of TIGS constructs.

^c^Significant deviation from median value of all bombarded TIGS constructs (1-sample *t*-test, α 0.05, one-tailed).

^d^Test for significant over-representation of specific gene groups associated with a TIGS effect, compared to the set of randomly selected genes (Fisher’s exact, one-tailed).

^e^Confidence interval for resistance QTL on chromosome 5H (peak-marker LOD -1).

### NHR screening by TIGS

A total of 692 TIGS constructs targeting 631 genes and corresponding to 468 (68%) pathogen-regulated transcripts were bombarded into barley epidermal cells followed by challenge inoculation with *Bgt* (Table 1). TIGS of 44 candidate genes caused enhanced *Bgt* haustorium formation, and these were repeated in a total of at least five independent experiments. As shown in Table 3 the NHR screening resulted in the identification of 10 RNAi constructs that significantly enhanced non-host susceptibility, therefore presumably targeting genes required for resistance to the non-adapted *Bgt* fungus (*Rnr1-10*, for required for non-host resistance 1-10). By applying multiple-testing correction (FDR 0.1) to the significance threshold α four constructs targeting *Rnr1*, *Rnr3*, *Rnr5*, and *Rnr9* remained with a significant effect. However, two of the RNAi constructs without FDR-corrected significant *P* value targeting *Rnr6* and *Rnr8* gave rise to stable transgenic plants with clearly enhanced susceptibility to *Bgt* suggesting that the applied error 1 correction was too stringent (Douchkov *et al.*, to be published elsewhere). The TIGS construct with the most significant effect targeting *Rnr3*, which encodes the syntaxin protein Hv-SNAP34, had been reported before and has been used as positive control since then. Barley cv. Maythorpe was chosen for the TIGS screening because it has a high degree of NHR to *Bgt* (0.1% susceptible epidermal cells) as compared to the derived, widely-used stiff-straw mutant cv. Golden Promise, which is a universally susceptible model genotype exhibiting a certain degree of non-host susceptibility to initial penetration attempts by *Bgt*. This might explain why the breakdown of NHR in Maythorpe caused by any TIGS construct was rather weak and did not exceed approximately 5% susceptible cells (Table 3). The largest effect was caused by silencing Hv-*SNAP34*. Besides Hv-*SNAP34* that was also found to be required in NHR of *A. thaliana* to *Bgh* [30], none of the *Rnr* genes had been identified in NHR screens of other plant species.

**Table 3:** **Identification of** ***Rnr*** **genes required for NHR of barley to** ***B. graminis*** **f.sp.** ***tritici***

**Clone ID** ^**a**^	**RNAi target gene**	**Description (Blast X)** ^**b**^	**Susceptible cells (%)**		
			**Mean ± SE**	***P*** ^**c**^	**α (FDR 0.1)** ^**d**^
	Positive ctr^e^		4.84 ± 0.57	*0.0001*	0.0023
	Negative ctr^f^		0.10 ± 0.04	---	
HM01A17	*Rnr1*	Nonclathrin coat protein γ 1	1.08 ± 0.33	*0.007*	0.0070
HO10B14	*Rnr2*	BAH domain protein	0.35 ± 0.17	0.029	0.0140
HO14B18	*Rnr4*	Endo-1.4-beta-glucanase	0.56 ± 0.18	0.005	0.0047
HO14H18	*Rnr5*	ARM Repeat protein	0.35 ± 0.19	*0.007*	0.0093
HO02M14	*Rnr6*	Cellulose-synthase like D2	1.29 ± 0.49	0.036	0.0163
HO15P19	*Rnr7*	EF Hand protein	1.15 ± 0.43	0.048	0.0256
HO27O23	*Rnr8*	Receptor-like kinase	0.74 ± 0.32	0.044	0.0233
HU02G09	*Rnr9*	Subtilisin-like protein	0.50 ± 0.23	*0.009*	0.0186
HW03O11	*Rnr10*	Stomatin-like protein	0.54 ± 0.30	0.044	0.0116

^a^EST clone ID deposited at NCBI that was used for generation of the TIGS construct.

^b^Blast X results with an E-value of lower than 10^-10^.

^c^Mann-Whitney test against empty-vector control (one-tailed). *P* values lower/equal than Benjamini-Hochberg-corrected significance threshold α (FDR 0.1) are highlighted in italics.

^d^Significance threshold with false-discovery rate (FDR) 0.1.

^e^Silencing of Hv-*SNAP34* (*Rnr3*; Douchkov *et al.* [23]).

^f^Empty-vector control pIPKTA30N.

### QR screening by TIGS

As outline above, QR is a quantitative trait depending on favorable allelic combinations at relevant QTLs. Therefore, by selecting an appropriate combination of barley genotype and *Bgh* isolate resulting in a moderate level of QR it should be possible to shift the interaction in both directions, that is, enhanced resistance or susceptibility, depending on the host genes or alleles introduced or silenced. In the TIGS experimental setup, the combination of cv Maythorpe and *Bgh* isolate CH4.8 was well suited for this purpose because the average level of initial haustorium formation, which served as readout of QR, was 0.1 to 0.2 per penetration attempt, thereby allowing to observe shifts it in both directions. A total of 1,014 TIGS constructs targeting 914 host genes were tested (Table 1). Those increasing the percentage of susceptible (haustoria bearing) bombarded cells by a factor of at least 1.5 compared to the empty-vector control or decreasing it by a factor of 2 or more were selected for four additional, independent bombardments (Additional file 1). To address potential off-target effects of these plus the 44 RNAi constructs selected for repeated bombardments in the NHR screen, we performed off-target prediction in the set of predicted high-confidence genes of barley [31] by using the si-Fi software (Lück *et al.*, in preparation; [32]). This resulted in the identification of 69% of the TIGS constructs with zero to only one predicted off-target (Additional file 2). Where off-targets were predicted, we performed a BlastX search among the intended target and the two most significant off-targets by emphasizing on those RNAi construct that gave rise to 2% to 10% off-target-matching siRNAs compared to the main target This focus on rather weak off-targets should have maximized the chance of finding non-paralogous genes that still might be hit by a sufficiently high number of siRNAs for silencing, As a result we identified 78% of paralogous off-targets from the same multigene-family whereas 9% off-target genes appeared to be non-paralogs. Therefore, the specificity of TIGS probably allowed at least discovery of relevant gene families in about 90% of the cases.

**Figure 1:**
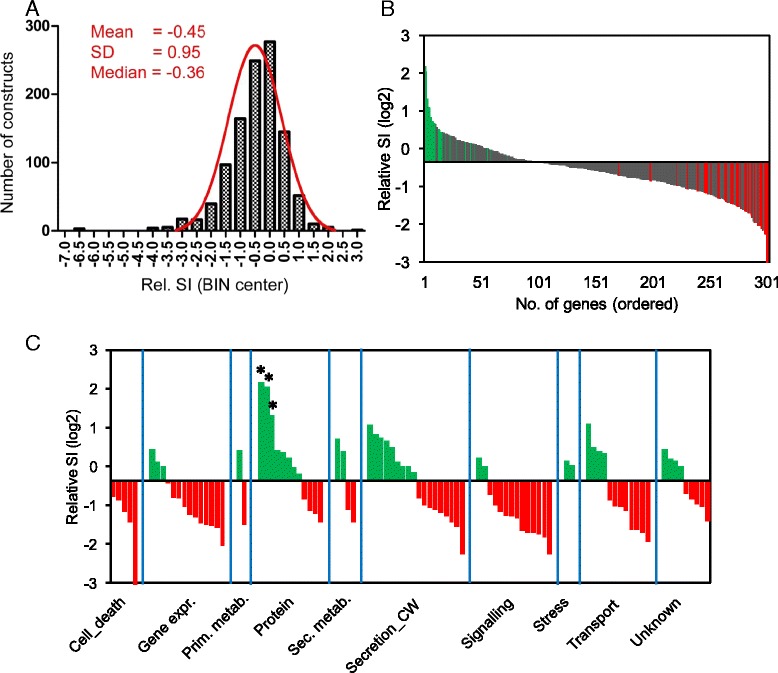
**TIGS of host genes affects the interaction of barley with the powdery mildew fungus** ***Bgh*** **. (A)** Distribution of log_2_-transformed relative SI values of all tested TIGS constructs in the first screening round. **(B)** Ordered mean values of TIGS effects of all constructs bombarded in at least five independent experiments, after the selection based on results from the first screening round. For completeness this overview includes 13 target genes to be reported later elsewhere. Green and red bars, significantly enhanced susceptibility and resistance, respectively (one-tailed *P* <0.05). **(C)** Ordered mean values of significant TIGS effects (*P* <0.05, one-tailed one-sample *t*-test) of all constructs bombarded in at least five independent experiments. The results are grouped according to a manual, broad functional-category assignment of corresponding target genes. CW, cell wall; expr., expression; metab., metabolism; Prim., primary; sec., secondary; secr., secretion; protein, protein translation, modification, or degradation; *, Polyubiquitin genes.

For statistical analysis the relative susceptibility index (SI, compared to the empty-vector control that was set to 0) were log_2_-transformed and compared to the log_2_-transformed median relative SI of the entire set of 1,014 bombarded constructs, which we expected - by its complexity - to be balanced with respect to positive or negative effects on *Bgh* infection, both types having been described before (see [17] and references cited therein). Unexpectedly the median value found was -0.36, possibly reflecting a sequence-non-specific stress effect of triggering the RNAi machinery in the bombarded cells. Out of 288 RNAi target genes silenced in repeated experiments, 44 affected the relative SI to *Bgh* in a statistically significant manner after multiple-testing correction (*P* <0.05 and FDR 0.1). Without this correction, the number of genes with significant TIGS effects increased to 90. The additional 46 genes represent weaker candidates, which was taken into account by assigning them a lower TIGS score in the final meta-dataset (see below). The average relative SI values of the repeatedly bombarded TIGS constructs were plotted against the median value of all tested 1,014 constructs. This revealed more constructs reducing susceptibility in a statistically significant manner (Figure 1B, red bars) than enhancing it significantly (green bars) suggesting that a majority of the silenced host genes might be involved in negative control of stress responses rather than defense. It remains open if some of these susceptibility-related genes are also co-opted by Bgh to facilitate fungal accommodation. To address the alternative possibility that certain constructs caused cell damage or death, which would also have prevented haustoria formation, by silencing genes with essential housekeeping function we tested all resistance-enhancing constructs in a cell-death assay, as reported [33-35]. This assay is based on reduced anthocyanin accumulation after induction of the pathway by transiently expressed *C1* and *b-Peru* transcription factors with or without co-bombarded TIGS constructs. Cellular stress was further increased by inoculating leaf segments with *Bgh* 3 days after co-bombardment of anthocyanin-inducing plus TIGS constructs (Additional file 3). However, no correlation between the strength of the resistance-enhancing and cell-death inducing effect was found leading to the conclusion that many of the strongly resistance-enhancing constructs may indeed target susceptibility - or accommodation - factors of the host. The only construct clearly reducing anthocyanin accumulation in repeated experiments targeted the housekeeping TCA-cycle enzyme ATP citrate lyase (arrow), besides two lethal positive-control constructs targeting polyubiquitin genes [33].

We compared the percentage of TIGS constructs with a significant effect on haustoria formation between the different groups of genes and found the lowest value of 2.3% to be associated with randomly selected genes whereas it was approximately 2 to 9 times higher in the defense-related groups selected based on background knowledge (Table 2). The corresponding increase in the TIGS hit rate was statistically significant for targets encoding cellulose synthase-like proteins (Csls), RLKs, and SWEET-like sugar transporters.

### Target transcript regulation

**Figure 2:**
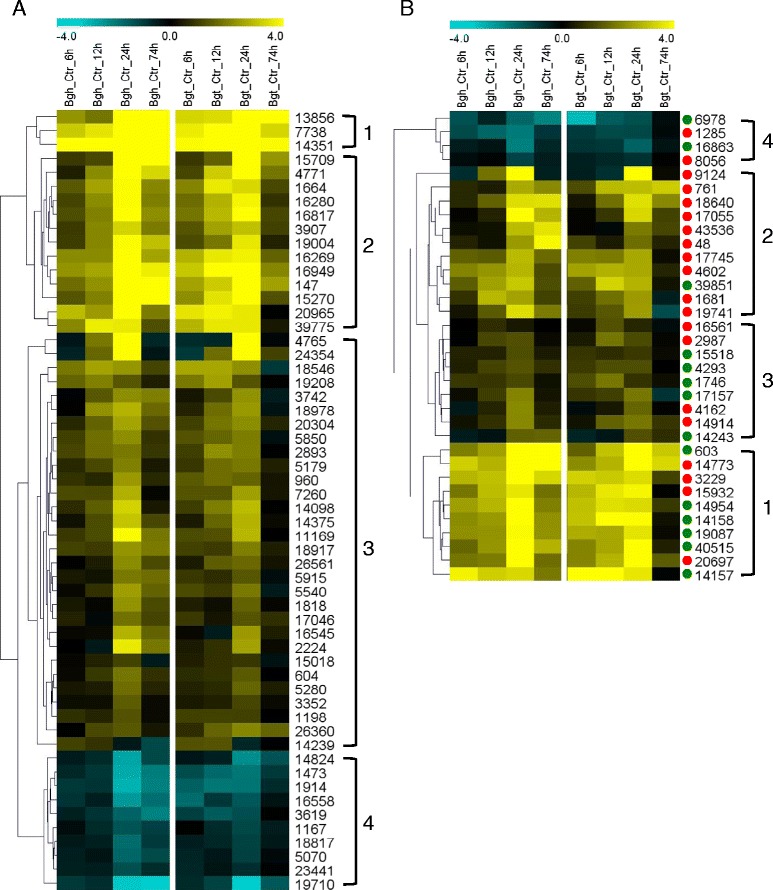
**Differential transcript regulation of target genes with and without significant TIGS effect on QR.** Figure legend text. Transcript regulation in leaf epidermis under attack by *Bgh* (susceptible host interaction) or *Bgt* (resistant nonhost interaction) was analyzed from 6 to 74 h after inoculation by using an Agilent 44 K oligunucleotide array. Ninety-one candidate genes were significantly regulated (> two-fold regulation; FDR <0.05). Log_2_-transformed fold change data of *Bgh*- or *Bgt*-inoculated samples *versus* controls (Ctr) were subjected to hierarchical clustering with Euclidian distance and average linkage settings. Numbers to the right of the hierarchical clustering displays correspond to unigene numbers in HarvEST assembly #35. Numbered brackets correspond to clades of transcripts with similar regulation behavior. **(A)** Genes without significant TIGS effects. **(B)** Genes with significant (*P* <0.05, one-tailed) TIGS effects. Green dots, enhanced susceptibility; red dots, enhanced resistance.

Out of the 288 target genes for QR that we tested in repeated TIGS experiments 277 were represented as oligonucleotide probes on a custom 44 K Agilent transcript-profiling array [36], and 90 were found to be significantly regulated by *B. graminis* attack in peeled epidermal samples (Figure 2). Most of these transcripts were upregulated possibly because this type of regulation had been chosen as one of the input criteria for the TIGS screenings (Table 1). A larger fraction of transcripts from genes with a significant TIGS effect were found to be rapidly induced within 6 h after inoculation compared to those not affecting relative SI upon TIGS (Figure 2, clade 1 in panels A and B). On the other hand, neither of the two groups contained members with clear differential expression in susceptible host- *versus* immune non-host interactions. Remarkably, almost all RNAi-target genes associated with host susceptibtility, that is, enhancing resistance upon silencing, were found to be associated with upregulated transcripts supporting our speculation that these candidate genes might indeed encode *bona fide* host susceptibility factors that have become co-opted by the invading fungus. The transcriptional behavior of the putative susceptibility factors is also in line with the results showing that TIGS did not often trigger cell death *per se* (Additional file 3), which would have offered an alternative explanation to the SI-reducing effects. It is interesting to note that the rapidly upregulated clade 1 contained significantly more resistance-associated genes causing enhanced susceptibility upon TIGS than the more slowly regulated clade 2 having its regulation peak at 24 h after inoculation. when the first haustorium was established in susceptible cells (*P* = 0.0237; two-tailed Fisher’s exact test).

### Meta-data analysis for QR to Bgh

The significant effects obtained by TIGS revealed a number of potentially important candidate genes affecting susceptibility or QR to *Bgh*. Including phenotypic data from transient over-expression of 11 candidate genes, for which no TIGS data were obtained [37,38] (Additional files 1 and 4), yielded four additional candidates (U35_1790, U35_2091, U35_5202, and U35_15506) for the meta-dataset. For meta-data analysis the TIGS/over-expression data were combined with results from transcript profiling reported here or derived from [25,39], QTL co-localization [28], SNP- or gene haplotype-trait association, as well as copy number variation (CNV) from own research or from the public domain. The association-genetic data were obtained in three collections of spring and in one panel of winter barley genotypes either by re-sequencing of candidate genes [26] or, as shown in Additional file 5, by a genome-wide association scan using the iSelect 9 K SNP chip of llumina Co. (Sharma, 2012, PhD thesis, Martin-Luther University Halle-Wittenberg). The combination of all five criteria provided a meta-dataset allowing to assign an additive score for converging evidence (CE) to each of the TIGS target genes that was bombarded in repeated experiments (Additional file 1). Genes were assigned one scoring point each for TIGS or over-expression effects with one-tailed *P* value <0.05, for significant transcript regulation in powdery mildew-attacked leaves, for a map position inside the confidence intervals of QTLs for resistance to *Bgh*, for significant association of SNPs and/or gene haplotypes with resistance or susceptibility to *Bgh*, and for significant CNV. One additional score was assigned if a gene exhibited a more significant TIGS or over-expression effect (two-tailed *P* value <0.05), or if its transcript was significantly regulated by powdery mildew attack in leaf epidermal peels, which is the tissue directly attacked by powdery-mildew fungi. The maximum CE score of seven was not reached by any candidate gene while four genes obtained the very high score of six (Table 4). The high relevance in the barley-*Bgh* interaction of two of these genes encoding a chorismate synthase and a germin-like protein of subfamily 4 was confirmed by independent studies in barley and wheat [37,40,41].

**Table 4:** **Barley candidate genes with a high score of converging evidence for a role in QR to** ***Bgh***

**U35-contig no.** ^**a**^	**CG no.**	**Proposed function (BlastX)**	**Functional category**	**Chr** ^**b**^	**Position (cM)** ^**b**^	**Linkage map** ^**b**^	**Transcript regul. epidermis**	**Transcript regul. leaf**	**Rel. SI (%)** ^**c**^	**TIGS or OEX**	**Transcr. regul.**	**CNV**	**Colocal Meta- QTL** ^**d**^	**Ass_SNP_ hapl_ GWAS**	**CE score** ^**e**^
964	1	BAX-Inhibitor1	Cell & death	6H	51.6	Marcel_09_int	UP	UP	41.9	2	2	1	0	0	5
3589	2	Hv-Lsd1a	Cell & death	5H	43.1	BOPA_cons	NS	NS	49.9	2	0	1	1	1	5
16561	3	Hv-Mlo	Cell & death	4H	103.1	BOPA_cons	UP	UP	13.8	2	2	0	1	0	5
16942	4	Stomatin-like protein (Rnr10)	Cell & death	5H	44.2	9K_WGS_BxM	UP	UP	62.5	1	2	0	1	0	4
11820	5	AP2-EREBP transcription factor	Gene expr.	7H	120.4	9K_WGS_BxM	UP	-	39.0	2	2	0	0	-	4
15932	6	Hv-WRKY2	Gene expr.	7H	126.3	9K_WGS_BxM	UP	UP	44.6	1	2	0	0	1	4
383	7	Hv-WRKY21	Gene expr.	3H	54.4	BOPA_cons	NS	DOWN	41.5	2	1	0	1	-	4
4162	8	Hv-WRKY28	Gene expr.	5H	46.5	9K_WGS_BxM	UP	UP	56.7	2	2	0	0	0	4
43536	9	Hv-WRKY45	Gene expr.	3H	59.3	9K_WGS_BxM	UP	UP	39.4	2	2	0	1	-	5
2987	10	Os-WRKY68-like	Gene expr.	2H	92.3	9K_WGS_BxM	UP	NS	35.7	2	2	0	0	1	5
2705	11	Pre-mRNA splicing factor PRP38	Gene expr.	7H	33.0	Marcel_09_int	UP	NS	79.4	1	2	0	1	0	4
16863	12	6-Phosphogluconolactonase 2	Prim. metab.	2H	56.3	BOPA_cons	DOWN	DOWN	142.9	1	2	0	1	-	4
604	13	Alpha/beta hydrolase	Prim. metab.	4H	126.1	Marcel_09_int	UP	UP	72.2	0	2	0	1	1	4
5070	14	Short chain dehydrogen/reductase	Prim. metab.	5H	41.6	9K_WGS_BxM	DOWN	DOWN	173.9	0	2	1	1	-	4
15523	15	Stearoyl-ACP desaturase	Prim. metab.	2H	58.1	9K_WGS_BxM	UP	UP	78.5	0	2	1	1	-	4
3071	16	ARM repeat protein (Rnr5)	Protein	3H	95.4	BOPA_cons	UP	UP	49.0	2	2	1	0	0	5
17055	17	Nucellin-like aspartic protease	Protein	4H	48.5	BOPA_cons	UP	UP	41.4	1	2	0	1	0	4
19087	18	Subtilisin-like serine proteinase	Protein	3H	47.1	9K_WGS_BxM	UP	UP	137.4	2	2	0	1	-	5
13715	19	Ubiquitin	Protein	7H	104.1	9K_WGS_BxM	UP	NS	427.9	2	2	1	0	-	5
13712	20	Ubiquitin	Protein	5H	50.3	BOPA_cons	NS	NS	270.3	2	0	1	1	-	4
1746	21	4-Coumarate coenzyme A ligase	Sec. metab.	6H	59.8	Marcel_09_int	UP	UP	177.8	2	2	0	0	0	4
14914	22	Caffeic acid 3-O-methyltransferase	Sec. metab.	2H	89.9	Marcel_09_int	UP	UP	42.5	1	2	1	0	0	4
2091	23	Chorismate Synthase	Sec. metab.	4H	55.3	9K_WGS_BxM	UP	UP	72^f^	2	2	0	1	1	6
14239	24	Phenylalanine ammonia-lyase	Sec. metab.	2H	77.1	Marcel_09_int	UP	UP	95.5	0	2	1	1	-	4
14693	25	Calreticulin 1 or 2^g^	Secr. & CW	2H	151.4	BOPA_cons	UP	UP	101.9	1	2	0	1	1	5
17745	26	Golgi nucl.-sugar transporter	Secr. & CW	4H	65.8	9 K_WGS_BxM	UP	UP	56.5	2	2	0	0	-	4
6978	27	Hv-CslA11	Secr. & CW	3H	143.0	9 K_WGS_BxM	DOWN	DOWN	145.9	2	2	0	1	-	5
17157	28	Hv-CslD2 (Rnr6)	Secr. & CW	7H	3.8	9 K_WGS_BxM	UP	UP	216.3	2	2	1	1	0	6
14954	29	Hv-Ger4d (SOD)	Secr. & CW	4H	119.8	BOPA_cons	UP	UP	149.0	2	2	1	1	0	6
16280	30	Hv-Ger5a (SOD)	Secr. & CW	5H	97.4	Marcel_09_int	UP	NS	61.0	2	2	0	0	-	4
14157	31	Hv-Prx40	Secr. & CW	3H	81.2	Marcel_09_int	UP	UP	184.1	2	2	1	0	0	5
14158	32	Hv-Prx64	Secr. & CW	3H	81.2	Marcel_09_int	UP	UP	113.7	1	2	0	0	1	4
4293	33	Hv-SNAP34 (Rnr3)	Secr. & CW	2H	63.6	Marcel_09_int	UP	UP	179.8	2	2	0	1	0	5
16316	34	Diacylglycerol kinase	Signaling	2H	140.3	BOPA_cons	UP	UP	40.2	2	2	1	0	0	5
39894	35	Disease resistance protein Hcr2-0B	Signaling	-	-	-	UP	-	122.4	2	2	1	-	-	5
1818	36	OPDA reductase	Signaling	2H	64.2	BOPA_cons	UP	UP	68.8	0	2	0	1	1	4
15506	37	Receptor-like kinase (BAK-1)	Signaling	3H	142.7	Marcel_09_int	UP	UP	71.2^f^	2	2	0	0	0	4
18640	38	Receptor-like kinase (DUF26)	Signaling	5H	167.6	9 K_WGS_BxM	UP	UP	48.2	1	2	0	0	1	4
10720	39	Receptor-like kinase (DUF26)	Signaling	-	-	-	NS	UP	23.3	2	1	1	-	-	4
5850	40	Receptor-like kinase (lectin-like)	Signaling	5H	46.2	BOPA_cons	UP	UP	78.2	0	2	1	1	1	5
20697	41	Receptor-like kinase (lectin-like)	Signaling	7H	44.4	9 K_WGS_BxM	UP	UP	45.4	2	2	1	0	-	5
20304	42	Receptor-like kinase (lectin-like)	Signaling	2H	59.2	ZIPPER	UP	UP	60.5	0	2	1	1	-	4
26360	43	Receptor-like kinase (lectin-like)	Signaling	5H	46.2	CAPS_BxM	UP	NS	66.7	0	2	1	1	0	4
39885	44	Receptor-like kinase (LRR)	Signaling	-	-	-	NS	UP	32.6	2	1	1	-	-	4
16135	45	Triticum aestivum kinase (TAK)	Signaling	3H	6.8	9 K_WGS_BxM	NS	UP	52.1	2	1	1	0	-	4
16558	46	Glutathione S-transferase	Stress	4H	96.6	BOPA_cons	COMPL.	UP	101.2	0	2	1	1	0	4
1285	47	Sugar transporter (Os-SWEET2a)	Transport	1H	18.1	BOPA_cons	COMPL.	NS	40.8	1	2	0	1	0	4
2230	48	Charged MVB protein 5	Unknown	1H	0.2	9 K_WGS_BxM	UP	UP	86.7	0	2	1	1	-	4
14824	49	Hv-Ger2a	Unknown	2H	44.6	Marcel_09_int	DOWN	DOWN	58.4^f^	2	2	0	0	0	4
19741	50	Unknown protein	Unknown	7H	23.0	Marcel_09_int	UP	UP	51.8	2	2	1	1	0	6
1681	51	Unknown protein	Unknown	2H	136.2	Marcel_09_int	UP	UP	66.4	1	2	1	0	1	5

^a^HarvEST database.

^b^Map position derived from different mapping populations: 9 K_WGS_BxM, Barke x Morex population for Illumina 9 K SNP chip and WGS contig anchoring by POPSEQ; BOPA_cons, Barley OPA123-2008, consensus map for barley SNP genotyping deposited in GrainGenes database; CAPS_IxF, CAPS marker-based mapping in Igri x Franka DH population (Schweizer lab); Marcel_09_int, Consensus map, Barley, Integrated, Marcel 2009 deposited in GrainGenes database; ZIPPER, gene-order based map position using stringent sequence homology scores between cereal species.

^c^Relative susceptibility index caused by TIGS or transient over-expression, normalized to corresponding empty-vector controls.

^d^Map position lying between outmost flanking markers of meta-QTL (consisting of ≥3 overlapping QTL) for resistance to *Bgh*.

^e^Sum of scores assigned for: (1) TIGS or transient over-expression effect, (2) transcript regulation either in leaf epidermis or entire leaves, (3) significant copy number variation (CNV), (4) meta-QTL co-localization, and (5) SNP or haplotype association with QR to *Bgh*. More weight (2 CE scores) was assigned to significant TIGS or OEx effects after false-discovery correction (FDR 0.1), and to transcript regulation in the leaf epidermis (*versus* regulation in entire leaf samples).

^f^Effect of transient over-expression.

^g^Also regulates Ca^2+^ concentrations and is therefore also involved in signaling.

Ass, marker-trait association of SNP and/or gene haplotype in a candidate-gene approach or by genome-wide association (GWAS) analysis; Chr, chromosome; CE, convergent evidence; cM, centimorgan; CW, cell wall; expr., expression; metab., metabolism; prim, primary; sec., secondary; secr., secretion; SI, susceptibility index.

A more detailed discussion of individual candidate genes can be found in Additional file 6 provided online.

It has become increasingly clear in recent years that CNV resulting in gene sub-functionalization or enhanced transcript levels represents an important mechanism for rapid evolutionary adaptation of organisms to environmental changes. Outstanding in this respect are threats imposed by biotic stressors such as fungal pathogens because these engage (plant) hosts in co-evolutionary arm races. It is therefore not astonishing that stress-related genes, especially PRRs and NB-LRR-type resistance genes are over-represented among the ones with significant CNV profiles [42,43]. We therefore included data from a chip-based quantitative analysis of CNV in 14 genotypes of wild and cultivated barley using the cultivar Morex as reference in the meta-dataset [43]. Fifty-seven out of 292 genes (19.5%) tested in repeated TIGS or transient over-expression experiments exhibited significant CNV between Morex and one or several of the compared genotypes (Table 5). We tested if candidate genes for QR that are associated with high CE scores of 4 to 6 (after excluding the CE score for CNV itself) exhibit CNV more frequently than those with low scores of zero to 1. Indeed, we found a significantly higher fraction (33%) of CNV in genes with a high CE score compared to the low-scoring group (17%). Only about one-third of this difference could be explained by biased CNV occurrence in gene groups of barley belonging to different GO terms (see Additional file 6 and [43]) thus suggesting that the observed higher frequency of CNV among genes with high CE score might indeed be causally related to their proposed role in biotic stress responses. Next we tested a selection of genes with or without significant CNV for powdery-mildew-related differences in transcript abundance. This was done in a quantitative transcript analysis in the spring barley panel used for candidate-gene re-sequencing [27] and split into phenotypic bulks associated with susceptibility (bulk 1), penetration resistance (bulk 2), or late colony arrest that was frequently accompanied by darkly-pigmented spots visible to the naked eye (bulk 3). As shown in Additional file 7, 18 of 26 tested genes exhibited expression differences (p(*t*-test) <0.1) in *Bgh*-inoculated leaves between the susceptible and one or both of the resistant bulks. In some cases the absolute differences in transcript levels were small, which might reflect a rather small number of genotypes per bulk contributing to the difference. Approximately 88% and 61% of the selected genes with and without significant CNV, respectively, also exhibited differences in transcript levels between the phenotypic bulks. These results, although derived from a small sample of genes, indicate that CNV is a strong predictor of transcriptional differences upon pathogen stress, whereas absence of CNV does not exclude gene-regulatory effects that might be related to promoter polymorphisms (cis-effects) or genotype-dependent differences in upstream signaling (trans-effects). One example of causally linked CNV and differential transcript accumulation may be the dense, tandemly duplicated cluster of genes on chromosome 4HL encoding the secreted germin-like protein Hv-Ger4 with superoxide-dismutase activity. For this defense-related gene cluster we have hypothesized high transcript levels as evolutionary driving force shaping the locus [44,45].

**Table 5:** **Enhanced copy number variation among genes with high CE scores**

**Category**	**% CNV** ^**a**^	***P*** ^**b**^	**n**	**Reference**
All low-copy WGS contigs	14.9		115,003	Muñoz‐Amatriaín *et al.*, 2013 [43]
Tested genes (TIGS or OEX)	18.8		292	This report
TIGS/OEX effect NS	16.8		198	This report
TIGS/OEX effect significant	22.8	0.138	92	This report
Genes CE 0-1	17.3		98	This report
Genes CE 4-7	33.3	0.0374	39	This report

^a^Percentage of genes with significant copy number variation (CNV).

^b^ *P* value of Fisher’s exact test (one-tailed).

WGS, whole-genome shotgun.

**Figure 3:**
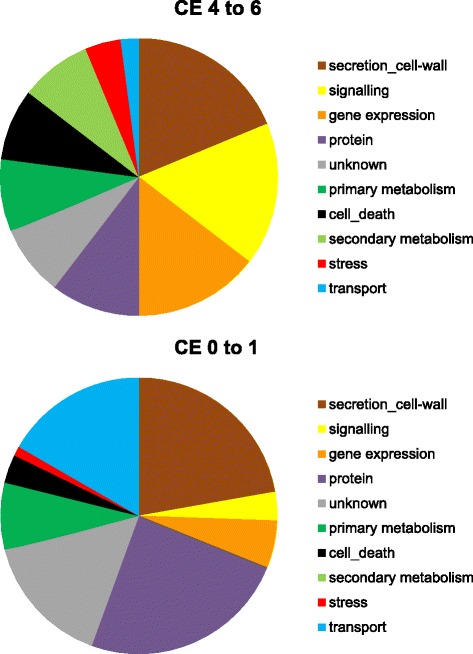
**Different functional-category distribution of genes with high CE score compared to low-scoring genes.** CE scores were attributed to candidate genes based on data from TIGS, transcript profiling, association mapping, and gene-QTL co-localization as described in Materials and Methods. Only genes with available data in four out of the five included datasets were taken into consideration. CE, convergent evidence. In total, 48 and 90 high- and low-scoring genes, respectively, were included in the analysis.

We found a different distribution of high *versus* low CE-scoring candidate genes among functional categories (Figure 3 and Additional file 1): Genes belonging to ‘signaling’ and ‘gene expression’ were strongly eQRiched in the set of high-scoring genes whereas a higher fraction of low scoring genes belonged to the categories ‘protein’ and ‘transport’. Overall, the difference in distribution among functional categories was significant (Chi-square, two-tailed *P* = 0.0003). Many highly CE-scoring candidate genes in the categories ‘signaling’ or ‘gene expression’ encode for RLKs or WRKY transcription factors. Thus, host factors involved in PAMP perception, transduction of corresponding signals, and execution of transcriptional programs appear indeed to be relevant for QR of barley to powdery mildew attack, in line with an eQRichment of RLK- and WRKY factor-encoding barley genes, respectively, inside meta-QTLs for resistance against powdery mildew [9].

## Discussion

Quantitative resistance of barley to *Bgh* is mediated by many QTLs with small to moderate effect [7,9,46,47]. This implies at least as many (non) host genes to be relevant for the trait. Despite steep progress in physical and genetic mapping as well as sequencing of the barley genome [31], a map-based cloning approach to all these trait-determining genes would still be very laborious and time-consuming. The same complex mode of inheritance might be true for NHR to non-adapted powdery mildew, similar to what has been described in non-adapted rust interactions [48], but experimental data are still missing to substantiate this speculative scenario. As an alternative approach, high-throughput reverse-genetic screenings may yield larger numbers of candidate genes provided the chosen strategy keeps the risk of false discoveries reasonably low. Reverse-genetic approaches can span a range of focusing levels from hypothesis-driven testing on one or few candidate genes up to screening the whole gene space of an organism. Here we describe an intermediate strategy by pre-selecting larger gene groups based on transcript-profiling data, gene-family membership, or QTL mapping and by entering these into a TIGS screening pipeline for NHR and QR.

As a result from the two primarily phenotype-driven TIGS screens in barley for larger groups of candidate genes we identified 10 genes designated *Rnr1-10* for NHR to the wheat powdery mildew fungus *Bgt* and up to 90 genes for QR or host susceptibility to the barley powdery mildew fungus *Bgh*, with an overlap of three to four genes (depending on stringency of statistical analysis) affecting both types of interactions. The overlapping genes encode: (1) for the syntaxin Hv-SNAP34 (*Rnr3*) that is likely to interact with the Ror2 (for required for mlo resistance 2) syntaxin engaged in vesicle-to-target membrane fusions during pathogen-induced transcytosis; (2) for an armadillo-repeat (ARM) protein representing a partial copy of a U-box/ARM E3 ubiquitin ligase (*Rnr5*); (3) for the cellulose-synthase like protein Hv-CslD2 that appears to be involved in cell-wall based defense (*Rnr6*); and (4) for a stomatin-like protein 2 (SLP-2)-related protein (*Rnr10*) involved in stress-induced mitochondrial hyperfusion, touch sensation, and T-cell activation in human and animals [49-51]. All four candidates are also among the 52 high-scoring genes for QR lending further support for their importance in durable pathogen resistance (Table 4). While *Rnr3* and *Rnr6* represent transporting and cargo components, respectively, of vesicle-mediated targeted secretion, *Rnr5* and *Rnr10* are likely to be involved in intracellular regulation of protein turnover and cell death. The four overlapping genes affecting both NHR and QR point at PTI as common defense mechanism, but also at co-option in the susceptible host interaction because TIGS of *Rnr5* and *Rnr10* resulted in enhanced resistance. By contrast, silencing of the remaining six *Rnr* candidate genes did not significantly affect host QR. Because all six including *Rnr8* (an LRR domain-containing RLK) are conserved between barley and wheat, we favor the idea that their apparent non-host-specific function might be related to inefficient direct or indirect neutralization by effectors of the non-adapted *Bgt*. Because not all genes with significant TIGS effect in the QR screening were also tested in the (earlier) NHR screening, it may well be that we currently underestimate the fraction of commonly utilized barley genes for controlling attacks by adapted- as well as non-adapted powdery mildews. Overall, we found one previously described and three novel genes of barley functioning in host- as well as non-host interactions with *Bgh* and *Bgt*, besides six genes for NHR only that might have become largely neutralized by *Bgh* effectors.

**Figure 4:**
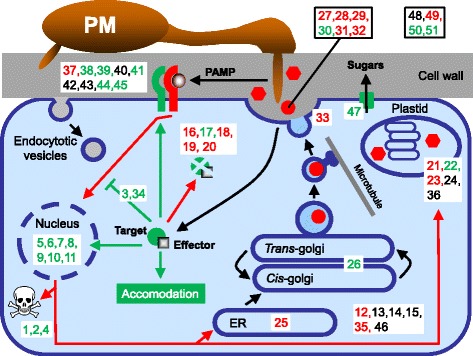
**Cellular mapping of candidate genes with high CE-score supporting a role in QR to** ***Bgh.*** Candidate genes with a CE score of at least 4 are shown. The corresponding gene numbers inside white boxes are derived from Table 4. Green and red labeling depicts susceptibility- and resistance-related gene function, respectively, as determined by TIGS. Black labeling indicates non-significant TIGS effect. Green and red arrows or symbols indicate susceptibility- or resistance-related interactions or molecules, respectively. Red circles, defense-related secreted proteins; red hexagons, defense-related cell-wall components including lignin-like material. The spoon-shaped structures at plant-cell membrane and the skull symbolize receptor-like kinases (PRRs plus potential co-receptors) and host cell death, respectively. ER, endoplasmic reticulum; PM, powdery mildew fungus.

In the TIGS screening for candidates of QR against *Bgh* an unexpectedly high fraction of 64% (35/55) resulted in significantly enhanced resistance suggesting that during the susceptible host interaction many barley genes support fungal accommodation (Figure 1B). A good proportion of these susceptibility-related gene candidates were associated with upregulated corresponding transcripts in attacked leaves, which might indicate their co-option by secreted *Bgh* effectors at the level of promoter activity or transcript stability. Three functional categories of genes were outstanding with respect to the bias for resistance-enhancing TIGS: (1) genes involved in cellular homeostasis and cell-death control; (2) transcriptional regulators including many WRKY factors; and (3) signaling genes including mostly RLKs (Figure 1C). The *P* values by Fisher’s exact test for deviation of resistance- *versus* susceptibility-enhancing effects from the null-hypothesis of 1:1 were found to be 0.12, 0.12, and 0.035, respectively, and thus only indicative for the first two categories. Nevertheless, this result proposes the genes within the three functional categories as potential targets to the identified CSEPs of *Bgh* [52]. It is interesting to note in this context that a positive mid-parent heterotic effect in a wheat F1 hybrid population for susceptibility to the wheat powdery mildew fungus was recently observed suggesting a disease-supporting effect of many genes with a dominant effect in the heterozygous state [53]. This behavior contrasted to negative mid-parent heterotic effects of the same hybrid population for susceptibility to leaf rust and *Septoria tritici* blotch. Generally, TIGS was found to be a reliable tool of gene discovery because we and others could reproduce TIGS-triggered changes in *Bgh* interaction phenotypes in stable transgenic RNAi plants or mutants of barley or *Arabidopsis* [23,30,34,54-56]. The TIGS results from the host screening with *Bgh* were combined with transcript profiling, association genetic, QTL co-localization as well as CNV results leading to a meta-dataset to which we assigned CE scores ranging from zero to 6. Out of 292 repeatedly bombarded genes entered into this analysis we identified 52 candidates with a CE score of at least 4 thus representing an initial inventory of genes with a proposed role in QR (Table 4). We tentatively mapped these onto cellular processes and compartments in a powdery mildew-attacked barley epidermal cell in order to get an impression of important defense- or susceptibility-related pathways (Figure 4). The 52 high-scoring candidate genes were also searched for available literature information with respect to plant-pathogen interactions (Additional file 8). This revealed 37 genes that have either been directly described in the interaction of barley or other plant species with microbial pathogens, or represent homologs and gene-family members of such functionally-characterized genes. A good proportion of these (78%) include known components of PTI or ETS pathways such as pattern recognition receptors (PRRs) encoded by RLK genes, WRKY transcription factors, the cell-death control factors Hv-*Mlo*, Hv-*Lsd1a*, and *Bax-inhibitor 1*, a member of the SWEET-family of sugar transporters, enzymes of the shikimate and phenylpropanoid plus oxidative pathways leading, for example, to cell-wall lignification [2,22,57-62]. It therefore appears that in the *Triticeae* crop plant barley QR indeed reflects the difference between PTI and ETS, as suggested in model plant systems [63] (Figure 4 and Additional file 8). Interestingly, genes involved in early steps of PTI (Figure 4, left side of the model) appear to be often involved in mediating susceptibility (TIGS results, highlighted by green gene numbers) whereas genes positioned further downstream of the pathway (right side of the model) are more likely to encode defense-related proteins (TIGS data, highlighted by red gene numbers). It has to be considered, though, that a large proportion of genes entered the TIGS QR screen because of their transcript-up-regulation upon pathogen attack or gene-family membership, which might have biased the distribution of functional categories among low and high CE-scoring genes. However, several multigene families including ABC transporters or class III peroxidases with clearly identified, important roles in QR or NHR did not show an increased frequency of TIGS effects among their members (Table 2). This finding argues against a strong family bias introduced by the gene-selection procedure and rather suggests that functional diversification among members of eukaryotic multigene families tends to be too complex for family-wide predictions about their involvement in a particular biological process. The non-predictability of physiological gene function is probably further pronounced in di- or tritrophic interactions between host plants and attacking parasitic organisms because these always reflect the current status of a highly dynamic and interaction-specific co-evolutionary arms race.

Among the high-scoring genes 15 have no record in the literature for being involved in plant defense or pathogen accommodation, thereby offering extensions to existing models of plant-pathogen interactions, at least as far as biotrophic fungal pathogens are concerned (Additional file 8). Some of those appear especially interesting: First, an aintegumenta-like AP2/EREBP transcription factor (QR. 5, U35_11820) was found to be upregulated in *Bgh*-attacked epidermis and to induce strong resistance when silenced. This type of transcription factor is associated with dividing meristematic tissue and might be a first lead into effector-triggered endo-reduplication in barley epidermis as recently suggested in a susceptible *A. thaliana*-powdery mildew interaction [64,65]. Second, a spliceosome component encoded by the PRP38-like gene U35_2705 might point at an important role of pre-mRNA processing to support fungal growth [66]. Third, an ARM-repeat protein encoded by U35_3071 (*Rnr5*) was identified as partial gene duplicate of an Os*-PUB15*-related E3-ubiquitin ligase. Deeper investigation suggested that this partial protein might represent a decoy for the E3-ligase targeted by a *Bgh*-encoded effector (Rajaraman and Schweizer, unpublished). Fourth, silencing of the transcriptionally upregulated *Rnr6* gene encoding Hv-CslD2, a member of the cellulose synthase-like (Csl) protein family, caused attenuation of QR and NHR (Tables 3 and 4). We therefore tested all available *Csl* unigenes of barley for a potential role in QR. This revealed Hv-*CslA11* as additional candidate with a high CE score. Several members of the Csl family are known to synthesize non-cellulosic cell-wall carbohydrates such as mixed-linked β-1-3:1-4-glucans during the build-up or modification of secondary cell walls while the enzymatic activity of others is still unclear [67]. Members of the CslA- and CslD-clades of the Csl protein family were shown or proposed to act as mannan synthases putting forward mannose-containing polysaccharides as potentially important for penetration resistance [68-70]. However, defense-related functions of mannan(s) in plants are currently not known [71]. A few more high-scoring candidate genes are discussed in Additional file 6.

## Conclusions

This study represents an initial, functional inventory of approximately 3% of the barley transcriptome for a role in NHR and QR against the powdery mildew pathogen. The discovered candidate genes support the idea that broad-spectrum, quantitative, and durable disease resistance in barley reflects to a large extent the difference of PAMP-triggered immunity minus effector-mediated host susceptibility. By extending the approach we expect more genes to be discovered in the future, which will require strong priorization for their labor-intensive validation in barley or related *Triticeae* crop plants. Meanwhile the ongoing in-depth analysis of the function of prioritized candidates will provide proof of concept for the approach of convergent evidence for the discovery of genes that are relevant to more durable forms of polygenically inherited pathogen resistance in barley.

## Materials and methods

### Plant and fungal material

For the TIGS screening 7-day-old seedlings of a spring barley cv. Maythorpe were used [72]. This genotype, from which the universal susceptible cv. Golden Promise was derived by γ-ray mutagenesis, proven to be well-suited for TIGS screenings in host as well as non-host interactions with powdery mildew fungi because it was fully resistant to the wheat powdery mildew *Bgt* while exhibiting a moderate level of QR to *Bgh*, thereby allowing to detect resistance- as well as susceptibility-enhancing TIGS effects in the host interaction. The mutagenesis leading to Golden Promise appeared to have affected multiple traits besides the initially targeted stiff-straw growth habit including salt tolerance, enhanced susceptibility to powdery mildew and high efficiency of *Agrobacterium*-mediated transformation [72]. Seedlings were grown in a plant incubator (Sanyo/Panasonic, address) at 20°C constant temperature, 50% rel. humidity and 16 h illumination (intensity level 5) by fluorescent tubes (OSRAM L36W/840).

For genome-wide association mapping of SNP markers with Bgh resistance, single-seed-derived 224 Genobar spring barley collection, plus 282 spring and 112 winter barley genotypes were used as described in [73] and [74]. For the detached leaf assay screening of resistance to Bgh, plants were grown in trays using disease free standard greenhouse conditions at 17°C to 20°C under long day conditions (16 h).

For the RT-qPCR-based transcript analysis of phenotypic bulks of barley differing in response to *Bgh*, single seed-derived lines of the following accessions were used: BCC1404, BCC1412, BCC1420, BCC1430, BCC1431, BCC1450, BCC1452, BCC1468, BCC1488, BCC1498, BCC423, BCC745, BCC888, BCC893, BCC903, HOR2800, HOR3941, HOR4060, BCC852, BCC1376, (susceptible bulk); HOR261, HOR728, HOR804, HOR842, HOR1036, HOR1457, HOR1506, HOR2543, HOR2591, HOR2932, HOR3270, HOR3271, HOR3537, HOR3726, HOR3988, HOR4021, HOR4408 (penetration resistant bulk); HOR214, HOR262, HOR303, HOR683, HOR736, HOR795, HOR800, HOR1159, HOR1379, HOR1468, HOR1647, HOR1873, HOR2573, HOR3041, HOR3075, HOR3983, HOR3984, HOR4400, (late resistant bulk). These plants were grown in the greenhouse with additional light (16 h) provided by sodium halogen lamps.

Plants of cv Vada used for the transcript profiling experiments were grown in a climate chamber at 19°C with 65% relative humidity (RH) during the night and 23°C with 45% RH during the 16 h photoperiod.

Inoculation experiments for TIGS or transcript profiling were performed using Swiss field isolate CH4.8 of *Bgh* or Swiss field isolate FAL 92315 of *Bgt.* For the detached leaf assay of the genome-wide association scan, the polyvirulent German *Bgh* isolates D12-12 and 78P were used as described [27]. Field data of *Bgh* resistance were derived from natural infection at IPK in 2009 and 2010.

### TIGS screenings

Target genes for TIGS were selected and analyzed based on sequence-contig information of the HarvEST database, barley 1.83 assembly #35 [75]. Putative off-target effects of TIGS constructs were predicted by using the si-Fi software (labtools.ipk-gatersleben.de) for finding all sequence matching putative siRNAfollowed by the application of an algorithm for siRNA guide strand selection as described [76]*.* TIGS Constructs were generated and transferred into barley leaf epidermal cells by particle bombardment followed by inoculation with *Bgh* and *Bgt* 3 and 4 days after bombardment, respectively, as described [23]. In the NHR screening, the number of susceptible cells carrying at least one *Bgt* haustorium was counted 48 h after inoculation. Constructs inducing at least four susceptible cells per bombardment were used for repeated bombardments resulting in a total of five independent biological replicates. Final results per TIGS construct were compared to the empty-vector control pIPKTA30 by using the Mann-Whitney non-parametric test. In the QR screening, transformed GUS-stained epidermal cells as well as haustoria-containing transformed (susceptible) cells were counted 48 h after inoculation. The susceptibility index (SI) was calculated by dividing the number of susceptible cells by the total number of transformed cells, followed by normalization to SI of the empty-vector control pIPKTA30N (rel. SI). Values of rel. SI were log_(2)_-transformed in order to normalize their distribution for statistical analysis by a one-sample *t*-test. This test was performed against the hypothetical relative susceptibility-index value ‘-0.355’ corresponding to the observed median of more than 1,000 RNAi constructs. The deviation from the control value of the empty pIPKTA30N vector (set to ‘0’) may reflect a weak and insert-non-specific side effect of triggering the cellular RNAi machinery.

### Cell-death assay

For the examination of cell death-inducing TIGS effects we performed particle co-bombardment of RNAi constructs pIPKTA30N_targetX (7 μg DNA/shot) with the *B-Peru*/*C1*-expression plasmid pBC17 (7 μg DNA/shot) triggering the anthocyanin biosynthetic pathway [54] and pUbiGUS (7 μg DNA/shot), as described by Dong *et al.* [33]. In this assay, cell death is reflected by a reduction of the GUS-normalized number of anthocyanin-accumulating cells.

### Transcript profiling

Seven-day-old barley plants of cv. Vada were inoculated with *Bgh* or *Bgt*, and the abaxial epidermis of inoculated primary leaves or from non-inoculated control leaves was peeled at 6 to 74 h after inoculation, as described [25]. Total, quality-controlled RNA was hybridized to a 44 K Agilent oligonucleotide array as described [36]. Single-channel array processing was utilized followed by data normalization with default parameters, and significant transcript-regulation events were determined by using GeneSpring GX (v11.5.1) software (Agilent technologies Inc). Transcripts were assumed to be significantly regulated if *P* values corrected for false-positive rate (FDR, Benjamini-Hochberg method) were less than 0.05 and if regulation factors between inoculated and corresponding control samples harvested in parallel exceeded 2.0. All quantile-normalized signal intensities of the analyzed candidate genes are shown in Additional file 9, and the raw data from the corresponding array slides were deposited at ArrayExpress (Accession E-MTAB-2916).

The RT-qPCR-based transcript analysis of phenotypic bulks of barley accessions differing in response to *Bgh* was performed by using Power SYBR® Green PCR Master mix kit (Applied Biosystems, Foster City, CA, USA) on an ABI 7900HT Fast Real-Time PCR system (Applied Biosystems, Foster City, CA, USA). Data were analyzed using the gene-testing standard-curve approach. Seven-day-old, greenhouse-grown (with additional light from sodium halogen lamps) seedlings were inoculated with *Bgh* at a density of approximately 10 to 30 conidia mm^-2^. Twelve hours after inoculation, total RNA was isolated [77]. RNA was treated with DNA-free™ Kit (Ambion, Austin, TX, USA) before cDNA synthesis. One microgram total RNA was used for cDNA synthesis using the iScript cDNA Synthesis Kit (Bio-Rad Co., Munich, Germany).

### Association mapping

Genotypic and phenotypic data of candidate-gene based association mapping by allele re-sequencing were derived from a previous study [27]. As significance threshold for SNP-trait or haplotype-trait association we selected the (-log_10_)*P* value of 3.0 in a general linear model including marker + trait + population structure + row number (covariant). Associations were calculated using the TASSEL software package.

Phenotypic data for genome-wide association mapping were derived from detached leaf assays of seedlings as described by Altpeter *et al.* [19] and from field data of spontaneous *Bgh* infection at IPK Gatersleben in 2009 and 2010. For field evaluation the percentage of leaf infection on a plot basis was scored and the Restriction Estimate of Maximum Likelihood (REML) implemented in Genstat software 14th edition (VSN International, Hemel Hempstead, UK) was used to generate means over the years. For the detached leaf assay screen, two polyvirulent Bgh isolates ‘D12-12’ and ‘78P’ were used, as described in [27]. Barley genotypes were only scored as resistant if the infection did not exceed rating class 1 [78] with either Bgh isolate. Genotypic data for genome-wide association mapping were derived from the Illumina 9 K SNP chip of barley [79]. SNP marker-trait associations were calculated in TASSEL 2.1 [80] and a mixed-linear model using kinship from random markers was used to control population structure. Marker-trait associations were considered as significant if the (-log_10_)*P* value was larger than 3.0 per single SNP in the candidate gene or if it exceeded 2.0 in the candidate gene plus in at least two immediately adjacent genes (sliding window approach). Per gene, the most significant association derived from field- or detached leaf-assay data was used for assigning ‘0’ or ‘1’ AM scores (Additional file 5).

### Gene functional categories

Each gene was manually assigned one out of 10 broad functional categories because the use of existing binning systems such as Gene Ontology or MapMan resulted in a large proportion of non-assigned genes that - by hand-curated BlastX analysis - could often be assigned one of the widely defined functional categories.

### Meta-data analysis

Primary transcript-regulation data of this study, or obtained from the public domain, were used [25,26] (PlexDB, [81]). Transcripts were assumed to be significantly regulated if normalized signal intensities (inoculated *versus* control samples) exceeded 2.0 in at least one of the analyzed time points after inoculation, and if the Benjamini-Hochberg-corrected *P* value for the null hypothesis was lower than 0.05. Regulation events in epidermal peels and in leaf samples were assigned 2 and 1 CE scores, respectively. If a gene was significantly regulated both in epidermal and leaf samples a score of 2 was assigned.

Candidate genes were tested for co-localization with meta-QTL for powdery-mildew resistance as described [9]. Briefly, co-localization was assumed if the gene was positioned between the outmost flanking markers of meta-QTL for resistance to *Bgh* consisting of ≥3 overlapping QTL in the consensus linkage map ‘Marcel *et al.*, integrated, 2009’ (deposited in GrainGenes 2.0 database).

Copy-number variation of candidate genes was tested in a panel of 14 barley genotypes and by using a custom Comparative Genomic Hybridization array designed by Roche NimbleGen (Roche NimbleGen, Inc., Madison, WI, USA) that used 2.2 M contigs from a whole genome shotgun (WGS) assembly of barley cv. Morex [43]. For CNV assessment the expectation maximization algorithm was used to estimate the mixing proportion, mean, and variance associated with two predicted signal sub-distributions found within the tested genotype vs. Morex fragments. When the log_2_ signal ratio was positive, the variant was defined as ‘UpCNV’, while it was classified as ‘DownCNV/PAV’ when the ratio was negative ([43]).

## Additional files

Additional file 1:
**List of TIGS target genes selected for repeated bombardments with their corresponding meta-dataset leading to the CE score.**
Additional file 2:
**Off-target analysis of all TIGS constructs used in repeated bombardments.**
Additional file 3:
**Resistance- and cell-death enhancing TIGS effects are not correlated.**
Additional file 4:
**Effect of transient over-expression of candidate genes exhibiting expression differences between susceptible and** ***mlo*** **-resistant near-isogenic barley lines.**
Additional file 5:
**Summary of results from genome-wide association scan for genes affecting QR to Bgh.**
Additional file 6:
**More detailed discussion of several candidate genes with high CE scores according to Table** **4** **, plus additional references.**
Additional file 7:
**Summary of results from transcript quantification in phenotypic bulks of barley genotypes with different responses to Bgh attack.**
Additional file 8:
**Literature survey of 42 high-scoring candidate genes with respect to plant-pathogen interactions.**
Additional file 9:
**Primary data of 91 significantly regulated transcripts corresponding to TIGS target genes tested in repeated bombardments of the QR screening.**

## References

[1] Jones JDG, Dangl JL (2006). The plant immune system. Nature.

[2] Boller T, Felix G (2009). A renaissance of elicitors: perception of microbe-associated molecular patterns and danger signals by pattern-recognition receptors. Annu Rev Plant Biol.

[3] Boehm H, Albert I, Fan L, Reinhard A, Nuernberger T (2014). Immune receptor complexes at the plant cell surface. Curr Opin Plant Biol.

[4] Fan J, Doerner P (2012). Genetic and molecular basis of nonhost disease resistance: complex, yes; silver bullet, no. Curr Opin Plant Biol.

[5] Schulze-Lefert P, Panstruga R (2011). A molecular evolutionary concept connecting nonhost resistance, pathogen host range, and pathogen speciation. Trends Plant Sci.

[6] Tosa Y (1992). A model for the evolution of formae speciales and races. Phytopathology.

[7] Niks RE, Marcel TC (2009). Nonhost and basal resistance: how to explain specificity?. New Phytol.

[8] Kou YJ, Wang SP (2010). Broad-spectrum and durability: understanding of quantitative disease resistance. Curr Opin Plant Biol.

[9] Schweizer P, Stein N (2011). Large-scale data integration reveals colocalization of gene functional groups with meta-QTL for multiple disease resistance in barley. Mol Plant-Microbe Interact.

[10] St Clair DA (2010). Quantitative disease resistance and quantitative resistance loci in breeding. Annu Rev Phytopathol.

[11] Koeck M, Hardham AR, Dodds PN (2011). The role of effectors of biotrophic and hemibiotrophic fungi in infection. Cell Microbiol.

[12] Lauge R, Joosten MH, Haanstra JP, Goodwin PH, Lindhout P, De Wit PJ (1998). Successful search for a resistance gene in tomato targeted against a virulence factor of a fungal pathogen. Proc Natl Acad Sci U S A.

[13] Poland JA, Balint-Kurti PJ, Wisser RJ, Pratt RC, Nelson RJ (2009). Shades of gray: the world of quantitative disease resistance. Trends Plant Sci.

[14] Dowkiw A, Bastien C (2007). Presence of defeated qualitative resistance genes frequently has major impact on quantitative resistance to Melampsora larici-populina leaf rust in P.xinteramericana hybrid poplars. Tree Genetics Genomes.

[15] Glawe DA (2008). The powdery mildews: a review of the world’s most familiar (yet poorly known) plant pathogens. Annu Rev Phytopathol.

[16] Collins NC, Sadanandom A, Schulze-Lefert P, Bélanger RR, Bushnell WR, Dik AJ, Carver TLW (2002). Genes and molecular mechanisms controlling powdery mildew resistance in barley. The Powdery Mildews.

[17] Huckelhoven R (2007). Cell wall - associated mechanisms of disease resistance and susceptibility. Annu Rev Phytopathol.

[18] Wise RP, Lauter N, Szabo LJ, Schweizer P, Muehlbauer GJ, Feuillet C (2009). Genomics of biotic interactions in the triticeae. Genetics and Genomics of the Triticeae.

[19] Altpeter F, Varshney A, Abderhalden O, Douchkov D, Sautter C, Kumlehn J, Dudler R, Schweizer P (2005). Stable expression of a defense-related gene in wheat epidermis under transcriptional control of a novel promoter confers pathogen resistance. Plant Mol Biol.

[20] Ihlow A, Schweizer P, Seiffert U (2008). A high-throughput screening system for barley/powdery mildew interactions based on automated analysis of light micrographs. BMC Plant Biol.

[21] Douchkov D, Lück S, Baum T, Seiffert U, Schweizer P, Tuberosa R, Graner A, Frison E (2014). Microphenomics for interactions of barley with fungal pathogens. Advances in Genomics of Plant Genetic Resources.

[22] Huckelhoven R, Dechert C, Kogel KH (2003). Overexpression of barley BAX inhibitor 1 induces breakdown of mlo-mediated penetration resistance to Blumeria graminis. Proc Natl Acad Sci U S A.

[23] Douchkov D, Nowara D, Zierold U, Schweizer P (2005). A high-throughput gene-silencing system for the functional assessment of defense-related genes in barley epidermal cells. Mol Plant-Microbe Interact.

[24] Elliott C, Zhou FS, Spielmeyer W, Panstruga R, Schulze-Lefert P (2002). Functional conservation of wheat and rice Mlo orthologs in defense modulation to the powdery mildew fungus. Mol Plant-Microbe Interact.

[25] Zellerhoff N, Himmelbach A, Dong WB, Bieri S, Schaffrath U, Schweizer P (2010). Nonhost resistance of barley to different fungal pathogens is associated with largely distinct, quantitative transcriptional responses. Plant Physiol.

[26] Zierold U, Scholz U, Schweizer P (2005). Transcriptome analysis of mlo-mediated resistance in the epidermis of barley. Mol Plant Pathol.

[27] Spies A, Korzun L, Bayles R, Rajaraman J, Himmelbach A, Hedley PE, Schweizer P (2012). Allele mining in barley genetic resources reveals genes of race-nonspecific powdery mildew resistance. Front Plant Sci Plant-Microbe Interact.

[28] Aghnoum R, Marcel TC, Johrde A, Pecchioni N, Schweizer P, Niks RE (2010). Basal host resistance of barley to powdery mildew: connecting quantitative trait loci and candidate genes. Mol Plant-Microbe Interact.

[29] Douchkov D, Johrde A, Nowara D, Himmelbach A, Lueck S, Niks R, Schweizer P (2011). Convergent evidence for a role of WIR1 proteins during the interaction of barley with the powdery mildew fungus Blumeria graminis. J Plant Physiol.

[30] Collins NC, Thordal-Christensen H, Lipka V, Bau S, Kombrink E, Qiu JL, Huckelhoven R, Stein M, Freialdenhoven A, Somerville SC, Schulze-Lefert P (2003). SNARE-protein-mediated disease resistance at the plant cell wall. Nature.

[31] Mayer K, Consortium IBS (2012). A physical, genetic and functional sequence assembly of the barley genome. Nature.

[32] ***si*****-*****Fi*****is a Software for RNAi (RNA interference) off-target prediction.** [http://labtools.ipk-gatersleben.de/]

[33] Dong WB, Nowara D, Schweizer P (2006). Protein polyubiquitination plays a role in basal host resistance of barley. Plant Cell.

[34] Eichmann R, Bischof M, Weis C, Shaw J, Lacomme C, Schweizer P, Duchkov D, Hensel G, Kumlehn J, Huckelhoven R (2010). BAX INHIBITOR-1 is required for full susceptibility of barley to powdery mildew. Mol Plant-Microbe Interact.

[35] Pliego C, Nowara D, Bonciani G, Gheorghe DM, Xu R, Surana P, Whigham E, Nettleton D, Bogdanove AJ, Wise RP, Schweizer P, Bindschedler LV, Spanu PD (2013). Host-induced gene silencing in barley powdery mildew reveals a class of ribonuclease-like effectors. Mol Plant-Microbe Interact.

[36] Chen XW, Hedley PE, Morris J, Liu H, Niks RE, Waugh R (2011). Combining genetical genomics and bulked segregant analysis-based differential expression: an approach to gene localization. Theor Appl Genet.

[37] Zimmermann G, Baumlein H, Mock HP, Himmelbach A, Schweizer P (2006). The multigene family encoding germin-like proteins of barley. Regulation and function in basal host resistance. Plant Physiol.

[38] Johrde A, Schweizer P (2008). A class III peroxidase specifically expressed in pathogen-attacked barley epidermis contributes to basal resistance. Mol Plant Pathol.

[39] Moscou MJ, Lauter N, Caldo RA, Nettleton D, Wise RP (2011). Quantitative and temporal definition of the Mla transcriptional regulon during barley-powdery mildew interactions. Mol Plant-Microbe Interact.

[40] Hu PS, Meng Y, Wise RP (2009). Functional contribution of chorismate synthase, anthranilate synthase, and chorismate mutase to penetration resistance in barley-powdery mildew interactions. Mol Plant-Microbe Interact.

[41] Christensen AB, Thordal-Christensen H, Zimmermann G, Gjetting T, Lyngkjaer MF, Dudler R, Schweizer P (2004). The germinlike protein GLP4 exhibits superoxide dismutase activity and is an important component of quantitative resistance in wheat and barley. Mol Plant Microbe Interact.

[42] Zmienko A, Samelak A, Kozlowski P, Figlerowicz M (2014). Copy number polymorphism in plant genomes. Theor Appl Genet.

[43] Muñoz‐Amatriaín M, Eichten SR, Wicker T, Richmond TA, Mascher M, Steuernagel B, Scholz U, Ariyadasa R, Spannagl M, Nussbaumer T, Mayer KFX, Taudien S, Platzer M, Jeddeloh JA, Springer NM, Muehlbauer GJ, Stein N (2013). Distribution, functional impact, and origin mechanisms of copy number variation in the barley genome. Genome Biol.

[44] Druka A, Kudrna D, Kannangara CG, Von Wettstein D, Kleinhofs A (2002). Physical and genetic mapping of barley (Hordeum vulgare) germin-like cDNAs. Proc Natl Acad Sci U S A.

[45] Himmelbach A, Liu L, Zierold U, Altschmied L, Maucher H, Beier F, Muller D, Hensel G, Heise A, Schutzendubel A, Kumlehn J, Schweizer P (2010). Promoters of the barley germin-like GER4 gene cluster enable strong transgene expression in response to pathogen attack. Plant Cell.

[46] Miedaner T, Korzun V (2012). Marker-assisted selection for disease resistance in wheat and barley breeding. Phytopathology.

[47] Aghnoum R, Niks RE (2011). Transgressive segregation for very low and high levels of basal resistance to powdery mildew in barley. J Plant Physiol.

[48] Jafary H, Albertazzi G, Marcel TC, Niks RE (2008). High diversity of genes for nonhost resistance of barley to heterologous rust fungi. Genetics.

[49] Huang MX, Gu GQ, Ferguson EL, Chalfie M (1995). A stomatin-like protein necessary for mechanosensation in c-elegans. Nature.

[50] Tondera D, Grandemange S, Jourdain A, Karbowski M, Mattenberger Y, Herzig S, Da Cruz S, Clerc P, Raschke I, Merkwirth C, Ehses S, Krause F, Chan DC, Alexander C, Bauer C, Youle R, Langer T, Martinou JC (2009). SLP-2 is required for stress-induced mitochondrial hyperfusion. EMBO J.

[51] Christie DA, Mitsopoulos P, Blagih J, Dunn SD, St-Pierre J, Jones RG, Hatch GM, Madrenas J (2012). Stomatin-like protein 2 deficiency in T cells is associated with altered mitochondrial respiration and defective CD4^+^ T cell responses. J Immunol.

[52] Pedersen C, Ver Loren van Themaat E, McGuffin LJ, Abbott JC, Burgis TA, Barton G, Bindschedler L, Lu X, Maekawa T, Weßling R, Cramer R, Thordal-Christensen H, Panstruga R, Spanu PD (2012). Structure and evolution of barley powdery mildew effector candidates. BMC Genomics.

[53] Longin CFH, Gowda M, Muhleisen J, Ebmeyer E, Kazman E, Schachschneider R, Schacht J, Kirchhoff M, Zhao YS, Reif JC (2013). Hybrid wheat: quantitative genetic parameters and consequences for the design of breeding programs. Theor Appl Genet.

[54] Schweizer P, Pokorny J, Schulze-Lefert P, Dudler R (2000). Double-stranded RNA interferes with gene function at the single-cell level in cereals. Plant J.

[55] Schultheiss H, Dechert C, Kogel KH, Huckelhoven R (2002). A small GTP-binding host protein is required for entry of powdery mildew fungus into epidermal cells of barley. Plant Physiol.

[56] Hoefle C, Huesmann C, Schultheiss H, Bornke F, Hensel G, Kumlehn J, Huckelhoven R (2011). A barley ROP GTPase ACTIVATING PROTEIN associates with microtubules and regulates entry of the barley powdery mildew fungus into leaf epidermal cells. Plant Cell.

[57] Bueschges R, Hollricher K, Panstruga R, Simons G, Wolter M, Frijters A, Van DR, Van DLT, Diergarde P, Groenendijk J, Topsch S, Vos P, Salamini F, Schulze-Lefert P (1997). The barley Mlo gene: a novel control element of plant pathogen resistance. Cell.

[58] Dietrich RA, Richberg MH, Schmidt R, Dean C, Dangl JL (1997). A novel zinc finger protein is encoded by the Arabidopsis Lsd1 gene and functions as a negative regulator of plant cell death. Cell.

[59] Meng Y, Wise RP (2012). HvWRKY10, HvWRKY19, and HvWRKY28 regulate Mla-triggered immunity and basal defense to barley powdery mildew. Mol Plant-Microbe Interact.

[60] Hahlbrock K, Bednarek P, Ciolkowski I, Hamberger B, Heise A, Liedgens H, Logemann E, Nurnberger T, Schmelzer E, Somssich IE, Tan JW (2003). Non-self recognition, transcriptional reprogramming, and secondary metabolite accumulation during plant/pathogen interactions. Proc Natl Acad Sci U S A.

[61] Vellosillo T, Vicente J, Kulasekaran S, Hamberg M, Castresana C (2010). Emerging complexity in reactive oxygen species production and signaling during the response of plants to pathogens. Plant Physiol.

[62] Chen LQ, Hou BH, Lalonde S, Takanaga H, Hartung ML, Qu XQ, Guo WJ, Kim JG, Underwood W, Chaudhuri B, Chermak D, Antony G, White FF, Somerville SC, Mudgett MB, Frommer WB (2010). Sugar transporters for intercellular exchange and nutrition of pathogens. Nature.

[63] Truman W, de Zabala MT, Grant M (2006). Type III effectors orchestrate a complex interplay between transcriptional networks to modify basal defence responses during pathogenesis and resistance. Plant J.

[64] Nole-Wilson S, Tranby TL, Krizek BA (2005). AINTEGUMENTA-like (AIL) genes are expressed in young tissues and may specify meristematic or division-competent states. Plant Mol Biol.

[65] Chandran D, Inada N, Hather G, Kleindt CK, Wildermuth MC (2010). Laser microdissection of Arabidopsis cells at the powdery mildew infection site reveals site-specific processes and regulators. Proc Natl Acad Sci U S A.

[66] Blanton S, Srinivasan A, Rymond BC (1992). PRP38 encodes a yeast protein required for pre-messenger-Rna splicing and maintenance of stable U6 small nuclear-Rna levels. Mol Cell Biol.

[67] Wang LQ, Guo K, Li Y, Tu YY, Hu HZ, Wang BR, Cui XC, Peng LC (2010). Expression profiling and integrative analysis of the CESA/CSL superfamily in rice. BMC Plant Biol.

[68] Dhugga KS, Barreiro R, Whitten B, Stecca K, Hazebroek J, Randhawa GS, Dolan M, Kinney AJ, Tomes D, Nichols S, Anderson P (2004). Guar seed beta-mannan synthase is a member of the cellulose synthase super gene family. Science.

[69] Liepman AH, Wilkerson CG, Keegstra K (2005). Expression of cellulose synthase-like (Csl) genes in insect cells reveals that CslA family members encode mannan synthases. Proc Natl Acad Sci U S A.

[70] Doblin MS, De Melis L, Newbigin E, Bacic A, Read SM (2001). Pollen tubes of Nicotiana alata express two genes from different beta-glucan synthase families. Plant Physiol.

[71] Hrmova M, Burton RA, Biely P, Lahnstein J, Fincher GB (2006). Hydrolysis of (1,4)-beta-D-mannans in barley (Hordeum vulgare L.) is mediated by the concerted action of (1,4)-beta-D-mannan endohydrolase and beta-D-mannosidase. Biochem J.

[72] Forster BP (2001). Mutation genetics of salt tolerance in barley: an assessment of Golden Promise and other semi-dwarf mutants. Euphytica.

[73] Pasam RK, Sharma R, Malosetti M, van Eeuwijk FA, Haseneyer G, Kilian B, Graner A (2012). Genome-wide association studies for agronomical traits in a world wide spring barley collection. BMC Plant Biol.

[74] Tondelli A, Xu X, Moragues M, Sharma R, Schnaithmann F, Ingvardsen C, Manninen O, Comadran J, Russell J, Waugh R, Schulman AH, Pillen K, Rasmussen SK, Kilian B, Cattivelli L, Thomas WTB, Flavell AJ (2013). Structural and temporal variation in genetic diversity of european spring two-row barley cultivars and association mapping of quantitative traits. Plant Genome.

[75] **HarvEST, an EST database for crop plant species.** [http://www.harvest-web.org]

[76] Khvorova A, Reynolds A, Jayasena SD (2007). Functional siRNAs and miRNAs exhibit strand bias. Cell.

[77] Chomczynski P, Sacchi N (1987). Single-step method of Rna isolation by acid guanidinium thiocyanate phenol chloroform extraction. Anal Biochem.

[78] Schweizer P, Vallelianbindschedler L, Mosinger E (1995). Heat-induced resistance in barley to the powdery mildew fungus erysiphe-graminis F-Sp hordei. Physiol Mol Plant Pathol.

[79] Comadran J, Kilian B, Russell J, Ramsay L, Stein N, Ganal M, Shaw P, Bayer M, Thomas W, Marshall D, Hedley P, Tondelli A, Pecchioni N, Francia E, Korzun V, Walther A, Waugh R (2012). Natural variation in a homolog of Antirrhinum CENTRORADIALIS contributed to spring growth habit and environmental adaptation in cultivated barley. Nat Genet.

[80] TASSEL: **Software for association mapping of complex traits in diverse samples.** [http://www.maizegenetics.net] 10.1093/bioinformatics/btm308 17586829

[81] **MIAME/Plant Compliant Gene Expression Resources for Plants and Plant Pathogens.** [http://www.plexdb.org]

