# Supplementary material for: Discovery of genes affecting resistance of barley to adapted and non-adapted powdery mildew fungi
Source: Genome Biol. 2014 Dec 3;15(12):518. doi: 10.1186/s13059-014-0518-8 (PMC4302706; doi:10.1186/s13059-014-0518-8)
Supplement: Additional file 3: — Resistance- and cell-death enhancing TIGS effects are not correlated. [file 13059_2014_518_MOESM3_ESM.pdf]

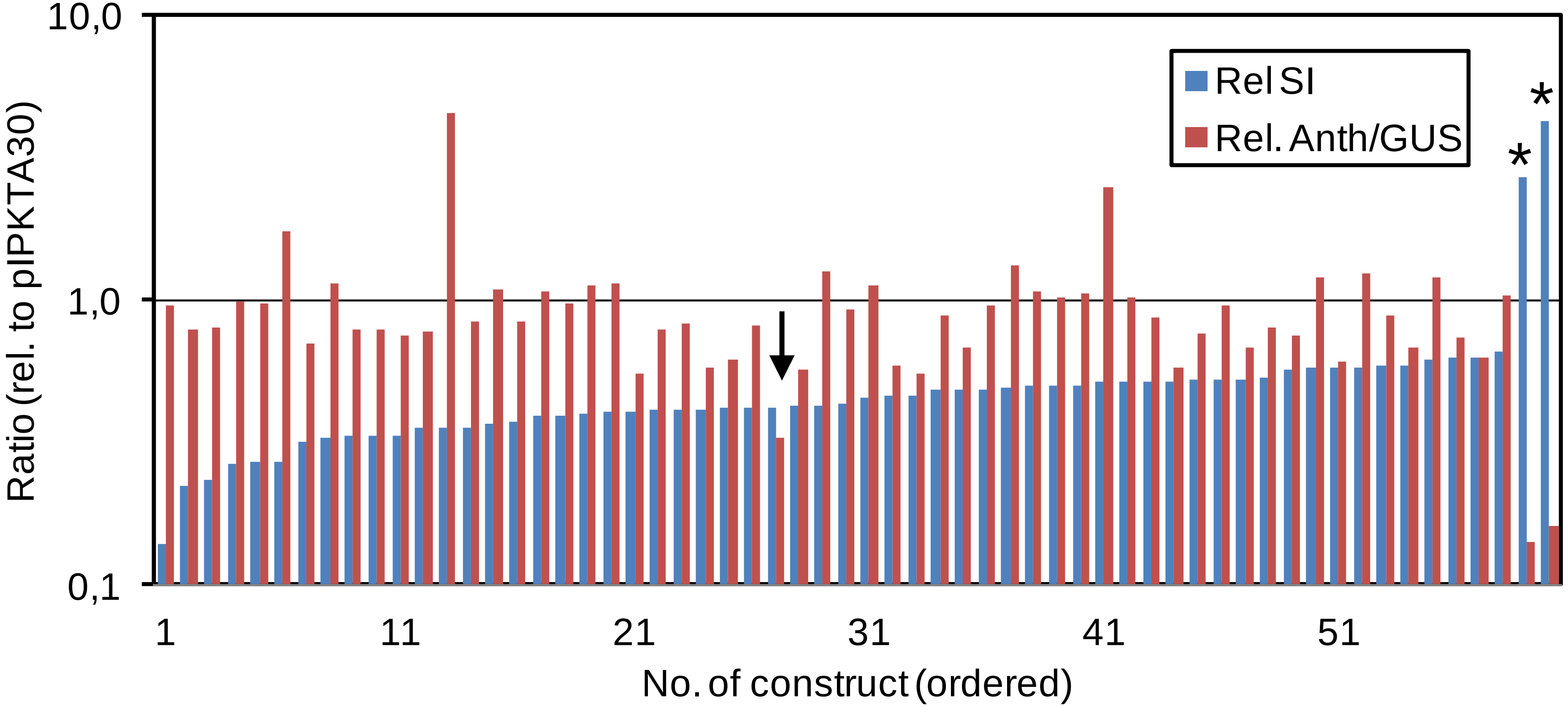

Additional Figure A1 - Resistance- and cell-death enhancing TIGS effects are not correlated.
All TIGS constructs that significantly ($p < 0.05$, 1-tailed) induced resistance to *Bgh* were also tested in a cell-death assay based on the reduction of transcription-factor induced anthocyanin accumulation during four days after bombardment. The number of anthocyanin-stained epidermal cells was normalized to *GUS* expression from a co-bombarded *GUS*-expression cassette. The arrow points at the cell-death inducing construct targeting an *ATP citrate lyase* gene. The asterisks mark two cell-death-inducing positive control genes encoding polyubiquitin.
