# Supplementary material for: Discovery of genes affecting resistance of barley to adapted and non-adapted powdery mildew fungi
Source: Genome Biol. 2014 Dec 3;15(12):518. doi: 10.1186/s13059-014-0518-8 (PMC4302706; doi:10.1186/s13059-014-0518-8)
Supplement: Additional file 4: — Effect of transient over-expression of candidate genes exhibiting expression differences between susceptible and mlo -resistant near-isogenic barley lines. [file 13059_2014_518_MOESM4_ESM.docx]

| U35 uni-  gene Nr. | Putative function (BlastX) | Rel. SI (log2)^a^ | p (t, 2-tailed) | n (Exp.) |
| --- | --- | --- | --- | --- |
| n.a. | Peroxidase TaPrx103^b^ | -1,09 ± 0,16 | 0,0023 | 5 |
| 14157 | Peroxidase HvPrx40^b^ | -0,48 ± 0,09 | 0,0053 | 5 |
| 2091 | Chorismate synthase | -0,49 ± 0,09 | 0,0068 | 5 |
| 15506 | Receptor-like kinase BAK-1 | -0,52 ± 0,13 | 0,0169 | 5 |

**Additional file 4:** Effect of transient over-expression of candidate genes on NR of barley to Bgh.

^a^Mean ± SEM from n independent experiments.

^b^Positive control construct (resistance enhancing, according to Johrde and Schweizer, 2008).
