# Supplementary material for: Discovery of genes affecting resistance of barley to adapted and non-adapted powdery mildew fungi
Source: Genome Biol. 2014 Dec 3;15(12):518. doi: 10.1186/s13059-014-0518-8 (PMC4302706; doi:10.1186/s13059-014-0518-8)
Supplement: Additional file 6: — More detailed discussion of several candidate genes with high CE scores according to Table 4 , plus additional references. [file 13059_2014_518_MOESM6_ESM.docx]

**Additional file 6: More detailed discussions plus additional References**

1. **More detailed discussion of copy-number variation (CNV) among candidate genes according to Table 5**

Previously, a significant over-representation of CNV was found to be associated with the stress-related GO terms “cell death” (GO:0008219) and “protein modification” (GO:0036211) (Muñoz‐Amatriaín et al., 2013). We therefore had to address the possibility that the difference in CNV between genes with high- and low CE scores might be caused by a biased distribution of functional categories (Figure 4). We analyzed the four most abundant functional categories among high- and low scoring candidates. Only one gene associated with a GO term descending from “cell death” was found whereas on average 13% of the genes were assigned a term descending from “protein modification”. The largest fraction (38%) of GO “protein modification”-derived genes was found in functional category “signaling” where it mostly accounted for protein phosphorylation activity of RLKs etc. Because category “signaling” was five times more abundant (20%) among genes with high CE score compared to low-scoring ones (4%), a bias for increasing CNV frequency simply by over-representation of GO “protein modification” had to be taken into account. We therefore estimated the maximum contribution of a biased GO-term abundance to CNV frequency to be in the range of 6% ((20%-4%)x38%), which is approximately three times less than the observed difference of 16% (33%-17%, Table 5).

1. **More detailed discussion of selected high CE-scoring candidate genes according to supplemental Table S4**

*6-Phosphocluconolactonase:*

Candidate gene Nr. 12 encodes 6-phosphocluconolactonase (*6-Pgl*) catalyzing the second enzymatic reaction of the plastidic oxidative pentose-phosphate pathway. The pathway was proposed as major source of NADPH reduction equivalents driving ROS production that are effective against biotrophic pathogens. Thus, escape from putative effector-mediated pathway repression might be an attractive approach for improving NR. The role of *6-Pgl* in plant defense was unknown until a recent report described homozygous 6-*pgl* knockout mutants of *Arabidopsis thaliana* exhibiting decreased cellular redox potential, probably by over-compensation, and increased resistance to the two biotrophic bacterial and oomycete pathogens *Pseudomonas syringae* and *Hyaloperonospora parasitica*, respectively ([Xiong et al., 2009](#_ENREF_28)). This is consistent with our observation that TIGS of a barley *6-Pgl* transcript increased susceptibility to *Bgh* and suggests that the 6-Pgl protein acts as a resistance factor against biotrophic pathogens. The observed down-regulation of *6-Pgl* transcripts in powdery mildew-attacked barley epidermis (Table 2 and Figure 3B) and in *P. syringae*-attacked *A. thaliana* plants ([Scheideler et al., 2002](#_ENREF_25)) might therefore reflect suppression of the defense-promoting effect of the encoded protein by successful pathogens.

*12-Oxophytodienoic acid reductase:*

The reduction of 12-oxophytodienoic acid (OPDA) by OPDA reductase is one of the committed biosynthetic steps of the stress hormone jasmonic acid accumulating in wounded- as well as pathogen-attacked plants and triggering – in its bioactive, isoleucine-conjugated form - defense responses mainly against herbivores and necrotrophic pathogens ([Staswick, 2008](#_ENREF_27); [Schaller and Stintzi, 2009](#_ENREF_24)). The tested barley *OPDA reductase* gene was co-localizing with a resistance *Meta*-QTL on chromosome 2H, exhibited SNP- as well as haplotype association with host response to *Bgh*, and was transcriptionally regulated by powdery-mildew attack. TIGS Of OPDA reductase showed a trend for enhanced resistance to *Bgh* although the effect was statistically not significant. Supporting this trend, TIGS of *allene oxide cyclase* (U35_1757) located upstream of *OPD reductase* in the jasmonic acid biosynthetic pathway did significantly induce resistance (supplemental Table S1) suggesting a rather disease-supporting effect of jasmonate signaling in the barley-Bgh interaction. Furthermore methyljasmonate levels remained low in leaves responding in a resistant manner to *Bgh* attack, and exogenously applied jasmonic acid did not induce the typical pattern of powdery mildew-induced PR proteins ([Schweizer et al., 1993](#_ENREF_26); [Kogel et al., 1995](#_ENREF_19)). Taken together, jasmonates might negatively interfere with defense-related signaling in *Bgh*-attacked epidermal cells, but their precise role including potential cross-talk to the defense-related stress hormone salicylic acid remains to be further addressed.

*Receptor-like kinases:*

RLKs constitute a highly complex multigene family in plants and fulfill developmental as well as stress-related functions ([Afzal et al., 2008](#_ENREF_1)). Biotic-stress-related RLKs are referred to as pattern recognition receptors (PRRs) and include FLS2 and EFR in *A. thaliana* or CERK1/CEBiP in rice recognizing bacterial flagellin, elongation factor EF-Tu or fungal cell-wall chitin fragments ([Boller and Felix, 2009](#_ENREF_3)). These PRRs residing in the plasmalemma are not acting alone but bind to co-receptors such as BAK1 (for BRI1-Associated Receptor Kinase 1) that also interacts with and regulates the brassinosteroid receptor BRI1 in *A. thaliana* ([Chinchilla et al., 2007](#_ENREF_8)). Here we report on three PRR-type RLKs containing N-terminal lectin-like- or cysteine-rich (DUF26) domains, and on a barley homologue of BAK1 as candidates with converging evidence for being relevant in barley/powdery mildew host interactions. Except for Hv-*Bak1* nothing is currently known about the biological function of the encoded RLK proteins or of close homologues in barley or other plant species. It also remains open at present if the Hv-Bak1 protein or any of the three identified RLKs cooperate for signal perception and PTI triggering. Anti-intuitively to the proposed function of RLKs as (co)-receptors for non-self molecules triggering PTI, silencing all three candidates with high CE scores increased NR suggesting them to represent susceptibility factors (Table 4). In barley a susceptibility-related DUF26-domain RLK was reported that, however, does not share significant sequence homology the the DUF26-domain protein (HarvEST contig U35_18640) described here ([Rayapuram et al., 2012](#_ENREF_23)). One of the possible ways how these RLKs could act as susceptibility factors is via effector-targeting, corruption and subsequent poisoning of multi-protein signaling complexes involved in PTI ([Faris et al., 2010](#_ENREF_14); [Zhang et al., 2010](#_ENREF_30); [Bouwmeester et al., 2011](#_ENREF_4); [Deslandes and Rivas, 2012](#_ENREF_9); [Feng et al., 2012](#_ENREF_15); [Zeng et al., 2012](#_ENREF_29)). Alternatively, effector targeting of these RLKs might pre-dispose cells to fungal accommodation, which would also suggest a role in healthy plants during cellular homeostasis, plant development or negative cell-death regulation. The observed significant over-representation of RLK-like genes exhibiting TIGS effects (Table 1) provides further support for their central role in determining the outcome of barley-powdery mildew host interactions.

*SWEET sugar transporters:*

A sugar transporter of the SWEET family caused resistance upon silencing. Therefore, as proposed in *A. thaliana* and rice for e.g. SWEET11, the Os-Sweet 2a-like protein identified here might be involved in providing nutrients to the fungus ([Chen et al., 2010](#_ENREF_7)). Astonishingly, this appears to be important already at a very early stage of host infection because TIGS reduced the formation of the first haustorium. Transporters of the SWEET family appear to be generally important for the final outcome of compatible barley/*Bgh* interactions because four out of 12 tested genes (33%) were associated with a significant TIGS effect resulting either in increased resistance or susceptibility (supplemental Table S1). The results also suggest a complex role of SWEET proteins during the interaction, with some members probably being involved in energy supply for epidermal defense rather than pathogen nutrition.

1. **REFERENCES CITED IN ADDITIONAL FILES**

**Afzal, A.J., Wood, A.J., and Lightfoot, D.A.** (2008). Plant receptor-like serine threonine kinases: Roles in signaling and plant defense. Molecular Plant-Microbe Interactions **21,** 507-517.

**Bhuiyan, N.H., Selvaraj, G., Wei, Y.D., and King, J.** (2009). Gene expression profiling and silencing reveal that monolignol biosynthesis plays a critical role in penetration defence in wheat against powdery mildew invasion. Journal of Experimental Botany **60,** 509-521.

**Boller, T., and Felix, G.** (2009). A Renaissance of Elicitors: Perception of Microbe-Associated Molecular Patterns and Danger Signals by Pattern-Recognition Receptors. In Annual Review of Plant Biology, pp. 379-406.

**Bouwmeester, K., de Sain, M., Weide, R., Gouget, A., Klamer, S., Canut, H., and Govers, F.** (2011). The Lectin Receptor Kinase LecRK-I.9 Is a Novel Phytophthora Resistance Component and a Potential Host Target for a RXLR Effector. Plos Pathogens **7**.

**Buschges, R., Hollricher, K., Panstruga, R., Simons, G., Wolter, M., Frijters, A., Van, D.R., Van, D.L.T., Diergarde, P., Groenendijk, J., Topsch, S., Vos, P., Salamini, F., and Schulze, L.P.** (1997). The barley Mlo gene: A novel control element of plant pathogen resistance. Cell **88,** 695-705.

**Caicedo, A.L.** (2008). Geographic diversity cline of R gene homologs in wild populations of Solanum pimpinellifolium (Solanaceae). American Journal of Botany **95,** 393-398.

**Chen, L.Q., Hou, B.H., Lalonde, S., Takanaga, H., Hartung, M.L., Qu, X.Q., Guo, W.J., Kim, J.G., Underwood, W., Chaudhuri, B., Chermak, D., Antony, G., White, F.F., Somerville, S.C., Mudgett, M.B., and Frommer, W.B.** (2010). Sugar transporters for intercellular exchange and nutrition of pathogens. Nature **468,** 527-U199.

**Chinchilla, D., Zipfel, C., Robatzek, S., Kemmerling, B., Nurnberger, T., Jones, J.D.G., Felix, G., and Boller, T.** (2007). A flagellin-induced complex of the receptor FLS2 and BAK1 initiates plant defence. Nature **448,** 497-U412.

**Deslandes, L., and Rivas, S.** (2012). Catch me if you can: bacterial effectors and plant targets. Trends in Plant Science **17,** 644-655.

**Dietrich, R.A., Richberg, M.H., Schmidt, R., Dean, C., and Dangl, J.L.** (1997). A Novel Zinc Finger Protein Is Encoded By the Arabidopsis Lsd1 Gene and Functions As a Negative Regulator Of Plant Cell Death. Cell **88,** 685-694.

**Dong, W.B., Nowara, D., and Schweizer, P.** (2006). Protein polyubiquitination plays a role in basal host resistance of barley. Plant Cell **18,** 3321-3331.

**Douchkov, D., Nowara, D., Zierold, U., and Schweizer, P.** (2005). A high-throughput gene-silencing system for the functional assessment of defense-related genes in barley epidermal cells. Molecular Plant-Microbe Interactions **18,** 755-761.

**Eckey, C., Korell, M., Leib, K., Biedenkopf, D., Jansen, C., Langen, G., and Kogel, K.H.** (2004). Identification of powdery mildew-induced barley genes by cDNA-AFLP: functional assessment of an early expressed MAP kinase. Plant Molecular Biology **55,** 1-15.

**Faris, J.D., Zhang, Z.C., Lu, H.J., Lu, S.W., Reddy, L., Cloutier, S., Fellers, J.P., Meinhardt, S.W., Rasmussen, J.B., Xu, S.S., Oliver, R.P., Simons, K.J., and Friesen, T.L.** (2010). A unique wheat disease resistance-like gene governs effector-triggered susceptibility to necrotrophic pathogens. Proceedings of the National Academy of Sciences of the United States of America **107,** 13544-13549.

**Feng, F., Yang, F., Rong, W., Wu, X.G., Zhang, J., Chen, S., He, C.Z., and Zhou, J.M.** (2012). A Xanthomonas uridine 5 '-monophosphate transferase inhibits plant immune kinases. Nature **485,** 114-U149.

**Hu, P.S., Meng, Y., and Wise, R.P.** (2009). Functional Contribution of Chorismate Synthase, Anthranilate Synthase, and Chorismate Mutase to Penetration Resistance in Barley-Powdery Mildew Interactions. Molecular Plant-Microbe Interactions **22,** 311-320.

**Huckelhoven, R., Dechert, C., and Kogel, K.H.** (2003). Overexpression of barley BAX inhibitor 1 induces breakdown of mlo-mediated penetration resistance to Blumeria graminis. Proceedings of the National Academy of Sciences of the United States of America **100,** 5555-5560.

**Johrde, A., and Schweizer, P.** (2008). A class III peroxidase specifically expressed in pathogen-attacked barley epidermis contributes to basal resistance. Molecular Plant Pathology **9,** 687-696.

**Kogel, K.H., Ortel, B., Jarosch, B., Atzorn, R., Schiffer, R., and Wasternack, C.** (1995). Resistance in barley against the powdery mildew fungus (Erysiphe graminis f.sp. hordei) is not associated with enhanced levels of endogenous jasmonates. European Journal of Plant Pathology **101,** 319-332.

**Meng, Y., and Wise, R.P.** (2012). HvWRKY10, HvWRKY19, and HvWRKY28 Regulate Mla-Triggered Immunity and Basal Defense to Barley Powdery Mildew. Molecular Plant-Microbe Interactions **25,** 1492-1505.

**Nadimpalli, R., Yalpani, N., Johal, G.S., and Simmons, C.R.** (2000). Prohibitins, stomatins, and plant disease response genes compose a protein superfamily that controls cell proliferation, ion channel regulation, and death. Journal of Biological Chemistry **275,** 29579-29586.

**Qiu, Y.J., Xi, J., Du, L.Q., Roje, S., and Poovaiah, B.W.** (2012). A dual regulatory role of Arabidopsis calreticulin-2 in plant innate immunity. Plant Journal **69,** 489-500.

**Rayapuram, C., Jensen, M.K., Maiser, F., Shanir, J.V., Hornshoj, H., Rung, J.H., Gregersen, P.L., Schweizer, P., Collinge, D.B., and Lyngkjaer, M.F.** (2012). Regulation of basal resistance by a powdery mildew-induced cysteine-rich receptor-like protein kinase in barley. Molecular Plant Pathology **13,** 135-147.

**Schaller, A., and Stintzi, A.** (2009). Enzymes in jasmonate biosynthesis - Structure, function, regulation. Phytochemistry **70,** 1532-1538.

**Scheideler, M., Schlaich, N.L., Fellenberg, K., Beissbarth, T., Hauser, N.C., Vingron, M., Slusarenko, A.J., and Hoheisel, J.D.** (2002). Monitoring the switch from housekeeping to pathogen defense metabolism in Arabidopsis thaliana using cDNA arrays. Journal of Biological Chemistry **277,** 10555-10561.

**Schweizer, P., Gees, R., and Mosinger, E.** (1993). Effect of Jasmonic Acid on the Interaction of Barley (Hordeum-Vulgare L) with the Powdery Mildew Erysiphe-Graminis F Sp Hordei. Plant Physiology **102,** 503-511.

**Staswick, P.E.** (2008). JAZing up jasmonate signaling. Trends in Plant Science **13,** 66-71.

**Xiong, Y.Q., DeFraia, C., Williams, D., Zhang, X.D., and Mou, Z.L.** (2009). Characterization of Arabidopsis 6-Phosphogluconolactonase T-DNA Insertion Mutants Reveals an Essential Role for the Oxidative Section of the Plastidic Pentose Phosphate Pathway in Plant Growth and Development. Plant and Cell Physiology **50,** 1277-1291.

**Zeng, L.R., Velasquez, A.C., Munkvold, K.R., Zhang, J.W., and Martin, G.B.** (2012). A tomato LysM receptor-like kinase promotes immunity and its kinase activity is inhibited by AvrPtoB. Plant Journal **69,** 92-103.

**Zhang, J., Li, W., Xiang, T.T., Liu, Z.X., Laluk, K., Ding, X.J., Zou, Y., Gao, M.H., Zhang, X.J., Chen, S., Mengiste, T., Zhang, Y.L., and Zhou, J.M.** (2010). Receptor-like Cytoplasmic Kinases Integrate Signaling from Multiple Plant Immune Receptors and Are Targeted by a Pseudomonas syringae Effector. Cell Host & Microbe **7,** 290-301.

**Zhang, L., Robbins, M.P., Carver, T.L.W., and Zeyen, R.J.** (1997). Induction of phenylpropanoid gene transcripts in oat attacked by Erysiphe graminis at 20 degrees C and 10 degrees C. Physiological and Molecular Plant Pathology **51,** 15-33.

**Zhang, W.D., Chen, J., Zhang, H.J., and Song, F.M.** (2008). Overexpression of a rice diacylglycerol kinase gene OsBIDK1 enhances disease resistance in transgenic tobacco. Molecules and Cells **26,** 258-264.

**Zimmermann, G., Baumlein, H., Mock, H.P., Himmelbach, A., and Schweizer, P.** (2006). The multigene family encoding germin-like proteins of barley. Regulation and function in basal host resistance. Plant Physiology **142,** 181-192.
