# Supplementary material for: Discovery of genes affecting resistance of barley to adapted and non-adapted powdery mildew fungi
Source: Genome Biol. 2014 Dec 3;15(12):518. doi: 10.1186/s13059-014-0518-8 (PMC4302706; doi:10.1186/s13059-014-0518-8)
Supplement: Additional file 8: — Literature survey of 42 high-scoring candidate genes with respect to plant-pathogen interactions. [file 13059_2014_518_MOESM8_ESM.docx]

**Additional File 8:** Candidate genes with high CE score for NR to *Bgh*.

| **U35 contig Nr.^a^** | **Gene Nr.** | **Proposed function (BlastX)** | **Functional category** | **Reference^b^** | **PTI, ETS^c^** |
| --- | --- | --- | --- | --- | --- |
| 964 | 1 | BAX-Inhibitor1 | Cell_death | Hueckelhoven et al, 2003 | YES |
| 3589 | 2 | Hv-Lsd1a | Cell_death | Dietrich et al, 1997 | YES |
| 16561 | 3 | Hv-Mlo | Cell_death | Buschges et al, 1997 | YES |
| 16942 | 4 | Stomatin-like protein (Rnr10) | Cell_death | Nadimpalli et al, 2000 |  |
| 11820 | 5 | AP2-EREBP transcription factor | Gene expression | none |  |
| 15932 | 6 | Hv-WRKY2 | Gene expression | Eckey et al, 2004 | YES |
| 383 | 7 | Hv-WRKY21 | Gene expression | Meng and Wise, 2012 | YES |
| 4162 | 8 | Hv-WRKY28 | Gene expression | Meng and Wise, 2012 | YES |
| 43536 | 9 | Hv-WRKY45 | Gene expression | Meng and Wise, 2012 | YES |
| 2987 | 10 | Os-WRKY68-like | Gene expression | Meng and Wise, 2012 | YES |
| 2705 | 11 | Pre-mRNA splicing factor PRP38 | Gene expression | none |  |
| 16863 | 12 | 6-Phosphogluconolactonase 2 | Primary metab. | Xiong et al, 2009 |  |
| 604 | 13 | Alpha/beta hydrolase | Primary metab. | none |  |
| 5070 | 14 | Short chain dehydrogenase/reductase | Primary metab. | none |  |
| 15523 | 15 | Stearoyl-ACP desaturase | Primary metab. | Song et al. 2013 |  |
| 3071 | 16 | ARM repeat protein (Rnr5) | Protein | Park et al. 2011 |  |
| 17055 | 17 | Nucellin-like aspartic protease | Protein | none |  |
| 19087 | 18 | Subtilisin-like serine proteinase | Protein | none |  |
| 13715 | 19 | Ubiquitin | Protein | Dong et al, 2006 | YES |
| 13712 | 20 | Ubiquitin | Protein | Dong et al, 2006 | YES |
| 1746 | 21 | 4-Coumarate coenzyme A ligase | Secondary metab. | Zhang et al, 1997 | YES |
| 14914 | 22 | Caffeic acid 3-O-methyltransferase | Secondary metab. | Bhuiyan et al, 2009 | YES |
| 2091 | 23 | Chorismate Synthase | Secondary metab. | Hu et al, 2009 | YES |
| 14239 | 24 | Phenylalanine ammonia-lyase | Secondary metab. | Bhuiyan et al, 2009 | YES |
| 14693 | 25 | Calreticulin 1 or 2 | Secretion_cell wall | Qiu et al, 2012 | YES |
| 17745 | 26 | Golgi nucl.-sugar transporter GONST3 | Secretion_cell wall | none |  |
| 6978 | 27 | Hv-CslA11 | Secretion_cell wall | none |  |
| 17157 | 28 | Hv-CslD2 (Rnr6) | Secretion_cell wall | none |  |
| 14954 | 29 | Hv-Ger4d (SOD) | Secretion_cell wall | Zimmermann et al, 2006 | YES |
| 16280 | 30 | Hv-Ger5a (SOD) | Secretion_cell wall | Zimmermann et al, 2006 | YES |
| 14157 | 31 | Hv-Prx40 | Secretion_cell wall | Johrde et al, 2008 | YES |
| 14158 | 32 | Hv-Prx64 | Secretion_cell wall | Johrde et al, 2008 | YES |
| 4293 | 33 | Hv-SNAP34 (Rnr3) | Secretion_cell wall | Douchkov et al, 2005 | YES |
| 16316 | 34 | Diacylglycerol kinase | Signalling | Zhang et al, 2008 |  |
| 39894 | 35 | Disease resistance protein Hcr2-0B | Signalling | Caicedo, 2008 |  |
| 1818 | 36 | OPDA reductase | Signalling | Raake et al. 2006 |  |
| 15506 | 37 | Receptor-like kinase (BAK-1) | Signalling | Chinchilla et al, 2007 | YES |
| 18640 | 38 | Receptor-like kinase (DUF26) | Signalling | Rayapuram et al. 2012 | YES |
| 10720 | 39 | Receptor-like kinase (DUF-26) | Signalling | Rayapuram et al. 2012 | YES |
| 5850 | 40 | Receptor-like kinase (lectin-like) | Signalling | Singh and Zimmerli, 2013 | YES |
| 20697 | 41 | Receptor-like kinase (lectin-like) | Signalling | Singh and Zimmerli, 2013 | YES |
| 20304 | 42 | Receptor-like kinase (lectin-like) | Signalling | Singh and Zimmerli, 2013 | YES |
| 26360 | 43 | Receptor-like kinase (lectin-like) | Signalling | Singh and Zimmerli, 2013 | YES |
| 39885 | 44 | Receptor-like kinase (LRR) | Signalling | Greeff et al. 2012 | YES |
| 16135 | 45 | TAK14-like protein kinase | Signalling | none |  |
| 16558 | 46 | Glutathione S-transferase | Stress | none |  |
| 1285 | 47 | Sugar transporter (OsSWEET2a-like) | Transport | Chen et al, 2010 | YES |
| 2230 | 48 | Charged multivesicular body protein 5 | Unknown | none |  |
| 14824 | 49 | Hv-Ger2a | Unknown | Zimmermann et al, 2006 |  |
| 15518 | 50 | Unknown protein | Unknown | none |  |
| 19741 | 51 | Unknown protein | Unknown | none |  |
| 1681 | 52 | Unknown protein | Unknown | none |  |

^a^HarvEST database.

^b^Same gene, close homologue or gene family discussed specifically with respect to plant-pathogen interactions in barley or another plant species.

^c^Gene (family) with described function in PTI or ETS pathways.
